# Beyond antimicrobials: multifunctional biomaterials for tissue regeneration

**DOI:** 10.3389/fbioe.2026.1917446

**Published:** 2026-10-27

**Authors:** Anna Mariano, Giuseppina Mariano, Francesca Liguori, Federica Zuppardi, Luigi Ambrosio, Maria Grazia Raucci

**Affiliations:** 1 Institute of Polymers, Composites and Biomaterials, National Research Council of Italy (IPCB-CNR), Naples, Italy; 2 School of Infection and Immunity, University of Glasgow, Glasgow, United Kingdom

**Keywords:** antimicrobial biomaterials, biofilm, biofilm-associated infections, immunomodulation, implant-associated infections, multifunctional biomaterials, phototherapy, regenerative medicine

## Abstract

Biomaterial-associated infections remain a major obstacle in regenerative medicine, wound healing, and implantology. While conventional anti-infective materials have mainly focused on preventing bacterial adhesion or delivering antimicrobial agents, infection control alone is often not enough to restore tissue function. In infected wounds and around implanted biomaterials, bacterial colonization, biofilm formation, immune dysregulation, oxidative stress, hypoxia, altered pH, and extracellular matrix degradation are tightly interconnected, creating a hostile microenvironment that delays or prevents regeneration. This has prompted a shift from passive antimicrobial surfaces toward multifunctional biomaterials designed to act as active therapeutic interfaces. Building on advances in antimicrobial and regenerative biomaterials, this review brings together perspectives on microbial control, microenvironment regulation, and host-tissue regeneration within a unified design framework. Particular attention is given to metal ion-releasing systems, ionic liquid-based materials, phototherapeutic platforms, nanozyme-based approaches, and structural and mechanical microenvironment engineering, including surface topography and porosity. By revisiting the classical “race for the surface” concept through a broader biological lens, this review highlights how successful biomaterial design must move beyond bacterial inhibition alone and coordinate microbial control with the restoration of a regenerative microenvironment. Finally, we outline current translational challenges, including long-term safety, dose control, manufacturability, environmental impact, and the need for more predictive models that evaluate both antibacterial efficacy and true tissue regeneration. Overall, next-generation antimicrobial biomaterials should not only eliminate infection, but also actively guide and orchestrate healing.

## Graphical Abstract

**Figure:**
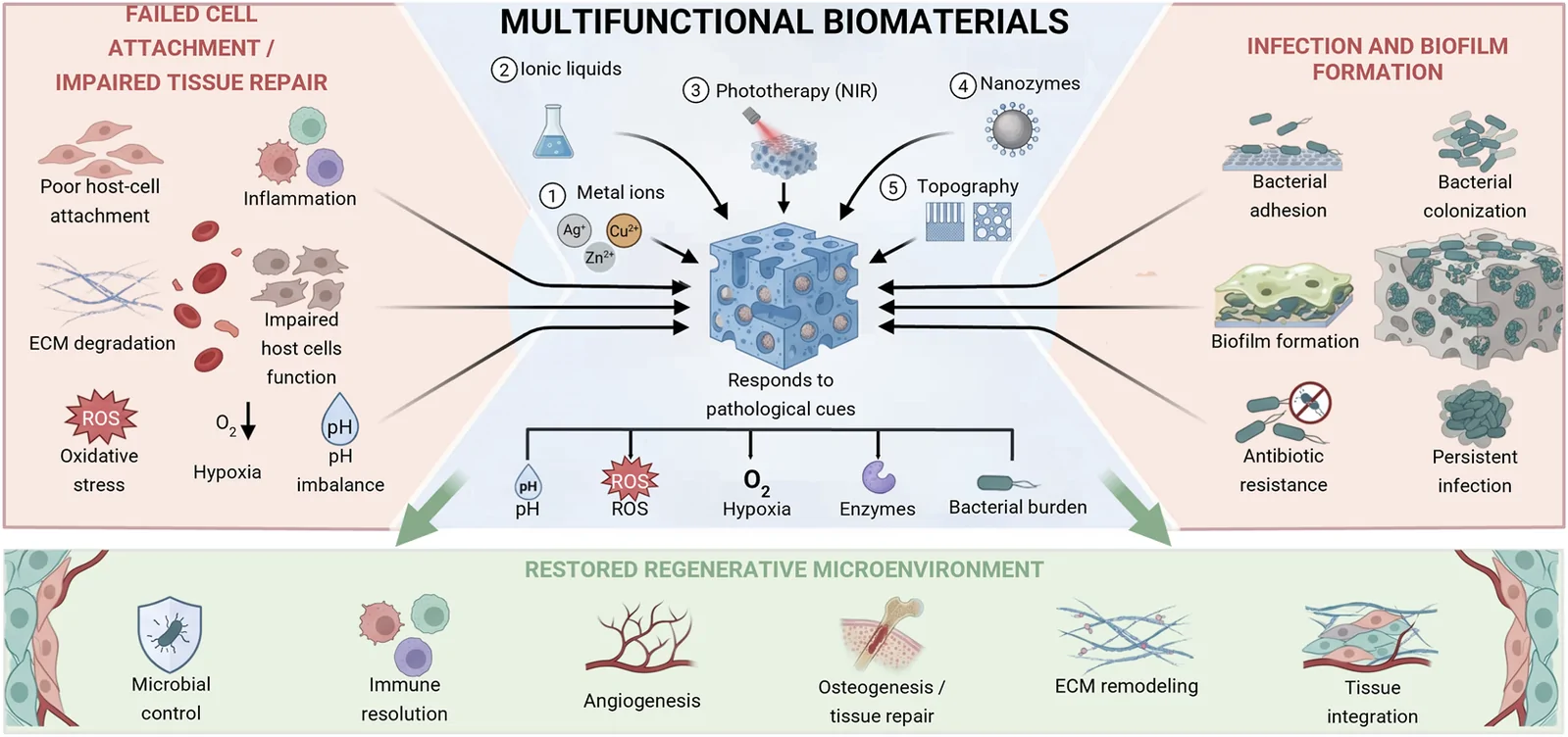
Multifunctional biomaterials integrate therapeutic components, including metal ions, ionic liquids, near-infrared phototherapy, nanozymes, and engineered surface topography, to address impaired host-cell attachment and tissue regeneration while inhibiting bacterial adhesion and biofilm formation. Through coordinated antimicrobial, immunomodulatory, angiogenic, extracellular-matrix-remodeling, and tissue-integration effects, they promote restoration of a regenerative microenvironment. Created in BioRender. Mariano, G. (2026) https://BioRender.com/uxmtw2n.

## 1 Introduction

Biomaterial-associated infections remain one of the most challenging complications in modern regenerative medicine, implantology, and wound care. The increasing use of medical devices, prostheses, tissue-engineered constructs, and advanced wound dressings has greatly improved clinical outcomes, yet it has also created new interfaces where microorganisms can adhere, colonize, and form biofilms. Once established, these infections are difficult to eradicate because bacteria embedded within biofilms are protected from immune clearance and display markedly reduced susceptibility to antimicrobial treatments (Trampuz and Zimmerli, 2005; Campoccia et al., 2006; Subbiahdoss et al., 2009; Li et al., 2020). In many cases, multiple bacterial species, and occasionally bacterial-fungal communities, may coexist on implant surfaces or within surrounding tissues, where interspecies interactions can alter biofilm architecture, virulence, immune responses, and susceptibility to antimicrobial treatment. This issue is further exacerbated by the global rise of antimicrobial resistance, which is progressively limiting the effectiveness of conventional antibiotic-based strategies (Ahmed et al., 2024; Habib et al., 2025). For decades, the design of anti-infective biomaterials has largely focused on preventing bacterial adhesion or killing pathogens through the incorporation of antibiotics, antiseptics, or antimicrobial ions. Although these approaches can be effective, they often rely on the release of bioactive agents, which may lead to burst release, short-lived activity, local toxicity, and the potential development of resistance. Moreover, infection control alone is not sufficient to ensure successful tissue regeneration. In infected wounds and around implanted biomaterials, bacterial colonization is closely intertwined with inflammation, oxidative stress, hypoxia, extracellular matrix degradation, and impaired host-cell function. Therefore, the next- generation of biomaterials must address infection and regeneration as interconnected biological processes rather than independent therapeutic targets. In this context, the classical concept of the “race for the surface” remains highly relevant, but it requires a broader interpretation. Successful biomaterial integration does not depend solely on whether host cells or bacteria first occupy the material interface. Rather, it depends on the ability of the material to actively shape the local microenvironment, prevent microbial dominance, modulate immune responses, and support constructive tissue remodeling (Trampuz and Zimmerli, 2005; Campoccia et al., 2006; Subbiahdoss et al., 2009; Li et al., 2020). Advanced biomaterials are therefore being engineered to combine antimicrobial activity with pro-regenerative functions, including immunomodulation, angiogenesis, osteogenesis, extracellular matrix organization, and real-time responsiveness to pathological cues (Miron et al., 2023; Kong et al., 2025; Luong and Luong, 2026). Recent progress in materials science has enabled the development of multifunctional platforms capable of integrating these diverse functions within a single system. Ion-releasing biomaterials, ionic liquid-based systems, phototherapeutic materials, nanozyme-based platforms, and other stimuli-responsive composites can be designed to act dynamically in response to infection-associated microenvironmental changes such as pH shifts, reactive oxygen species accumulation, enzymatic activity, hypoxia, or bacterial burden (Zhang et al., 2022; Thangudu and Su, 2025; Wei et al., 2025; Luong and Luong, 2026). These systems can provide localized and sustained antimicrobial activity while simultaneously restoring conditions favorable to tissue repair. Importantly, many of these materials move beyond passive protection, functioning as active therapeutic interfaces that interact with bacteria, immune cells, stromal cells, and the surrounding extracellular matrix.

Despite growing interest in antimicrobial and regenerative biomaterials, the combined relationships among microbial control, infection-associated microenvironment regulation, and host-tissue regeneration remain insufficiently integrated within a single design perspective. Addressing this gap is important for identifying common design principles that extend beyond the evaluation of antibacterial or regenerative efficacy alone.

This review discusses emerging multifunctional biomaterials that integrate antimicrobial performance with tissue-regenerative activity. The review is organized into three main sections. First, we examine the biological competition between microbial colonization and host tissue integration, highlighting how bacterial adhesion, biofilm formation, immune dysregulation, and microenvironmental disruption compromise regeneration. Second, we analyze major design strategies in modern multifunctional biomaterials, including metal ion-based systems, ionic liquid-containing materials, phototherapeutic platforms, nanozyme-based approaches and topography-mediated strategies. Attention is also given to how these systems simultaneously suppress infection and promote regeneration through controlled ion release, contact-active antimicrobial mechanisms, redox modulation, photothermal or photodynamic activation, immune regulation, and stimulation of angiogenic or osteogenic pathways. Finally, we consider current translational challenges and future directions for the development of clinically relevant infection-resistant regenerative biomaterials. By framing antimicrobial activity and tissue regeneration as interdependent processes, this review aims to highlight how multifunctional biomaterials are reshaping the future of infection-resistant regenerative medicine.

## 2 Multifunctional materials: A finishing line for the race for surface?

Biomaterials play a crucial role in modern medicine by restoring function, with common examples including prosthetic joints and heart valves. However, biomaterial-associated infections are a serious complication and are becoming more concerning as the use of implants and medical devices continues to rise. On average, biomaterials-associated infections occur in about 0.5%–6% of cases (Trampuz and Zimmerli, 2005; Campoccia et al., 2006), depending largely on the implant site, and are more frequent in trauma or revision surgeries (Trampuz and Zimmerli, 2005; Campoccia et al., 2006; Subbiahdoss et al., 2009; Li et al., 2020). The concept of the “race for the surface,” introduced by Gristina (1987), provides a useful framework for understanding implant-associated infections. This concept portrays a competition between bacterial adhesion and biofilm formation versus tissue integration on the implant surface. If tissue cells colonize the surface first, they create a protective layer that reduces susceptibility to bacterial infection. Conversely, if bacteria prevail, a biofilm rapidly forms, impairing tissue cell function (Figure 1). The implant surface may also trigger an inflammatory response that activates the immune system and can help limit bacterial colonization (Gristina, 1987; Subbiahdoss et al., 2009; Li et al., 2020). However, because microorganisms are often introduced during surgery or soon after implantation, they frequently gain an early advantage in this race (Li et al., 2020). During implantation, damage to epithelial or mucosal barriers can compromise the natural defenses of the body, creating an environment that favors microbial growth. This disruption allows pathogens to access deeper tissues and even the bloodstream. Additionally, biomaterials may release soluble substances and promote the formation of dense fibrous capsules around the implant (Capuani et al., 2022; Li R. et al., 2023; Kalashnikov et al., 2025). These capsules can act as physical barriers, limiting the ability of immune cells such as macrophages to reach the implant surface, thereby enabling pathogens to persist. Interactions between the biomaterial surface and surrounding biological media may also interfere with normal anti-infective processes, increasing the likelihood of infection spreading in adjacent tissues (Kannan et al., 2016). The selection of biomaterials is therefore critical, as their physicochemical properties strongly influence protein adsorption, microbial attachment, inflammation, and overall infection risk under physiological conditions.

**FIGURE 1:**
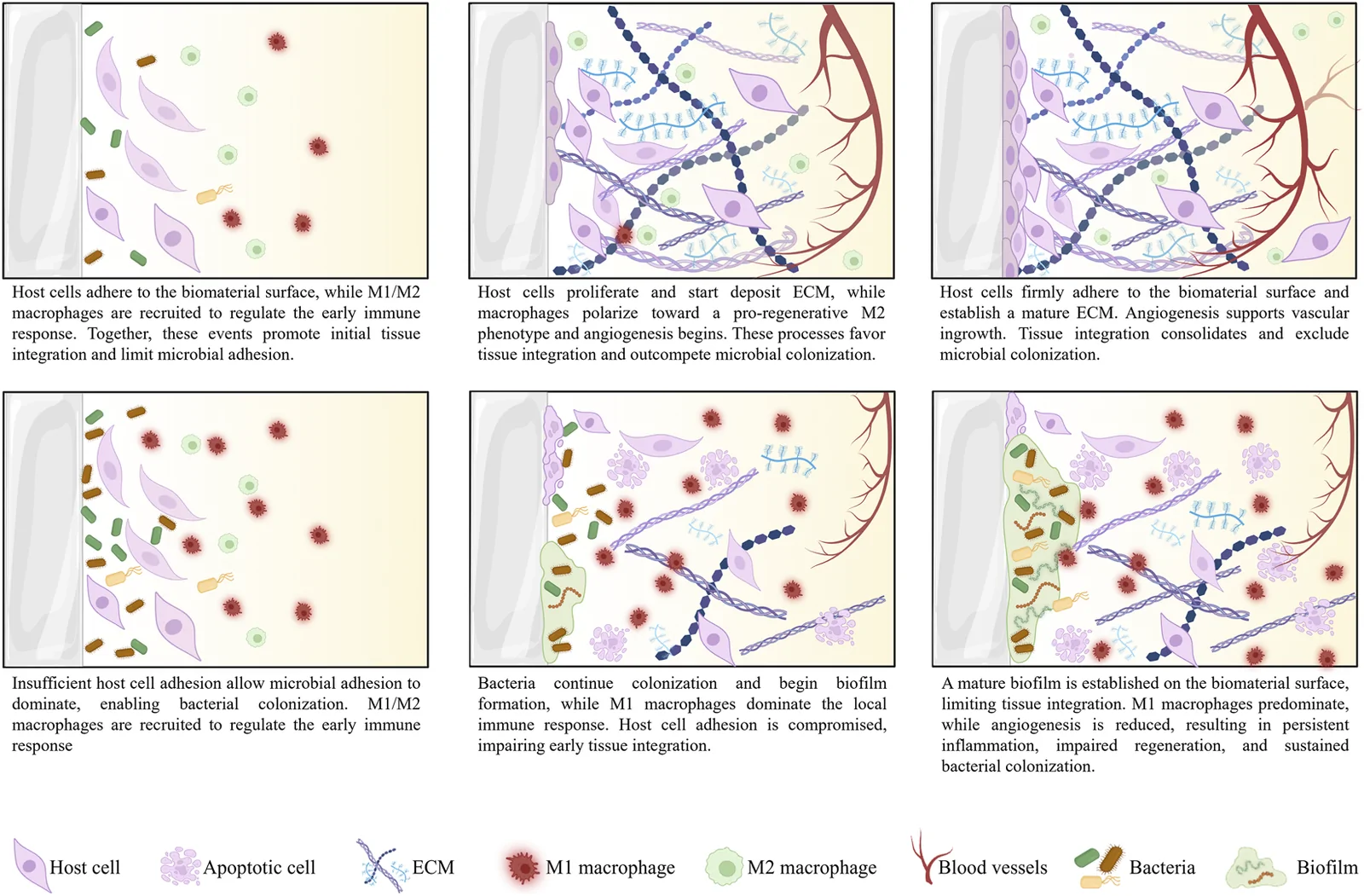
Race for the surface between host tissue integration and bacterial colonization on a biomaterial surface. The schematic illustrates the dynamic competition between host cells and bacteria following biomaterial implantation. In the favorable healing pathway, host cells rapidly adhere to the biomaterial surface, proliferate, and deposit ECM, while macrophages regulate the early immune response and progressively support tissue regeneration. Angiogenesis is initiated, promoting integration of the biomaterial with the surrounding tissue and limiting microbial adhesion. In the infection pathway, bacterial adhesion outcompetes host cell attachment, leading to progressive surface colonization and biofilm formation. As the biofilm matures, host cell adhesion and ECM deposition are compromised, angiogenesis is reduced, and a persistent pro-inflammatory macrophage response is sustained, ultimately impairing tissue integration and favoring chronic infection. Created in BioRender. Mariano, G. (2026) https://BioRender.com/p3k7o5s.

### 2.1 Tissue regeneration

Host cell adhesion to an implanted biomaterial is a tightly regulated, multi-step process that sets the trajectory for regeneration. Within seconds of implantation, the surface is conditioned by a layer of plasma proteins (e.g., fibronectin, fibrinogen, and albumin), whose composition and conformation are dictated by the material’s chemistry, charge, and topography (the Vroman effect) (Mitra, 2020; Richter-Bisson and Hedberg, 2025). These proteins present integrin-binding motifs that enable attachment of endothelial cells, fibroblasts, mesenchymal stromal cells, and immune cells, through receptors such as β1- and αv-containing integrins. Engagement of these receptors drives focal adhesion assembly (via FAK/Src signaling), actin cytoskeletal organization, and activation of downstream pathways (e.g., PI3K/Akt, MAPK) that regulate cell survival, proliferation, and migration (Pang et al., 2023; Tollabi et al., 2024). As cells spread and form a confluent layer, they begin depositing their own extracellular matrix, replacing the provisional protein layer with a more stable, tissue-specific matrix that supports long-term integration (Figure 1) (Chen Z. et al., 2024). This early adhesive phase is closely coupled with immune modulation and vascularization. Properly adhered cells can attenuate excessive inflammatory signaling by modulating macrophage polarization toward pro-regenerative phenotypes, while endothelial cell adhesion and migration initiate neovascularization, ensuring oxygen and nutrient delivery to the developing tissue. However, if adhesion is weak or delayed, cells fail to establish a continuous layer, leading to persistent exposure of the biomaterial surface (Trampuz and Zimmerli, 2005; Li et al., 2020). This not only sustains inflammatory cell recruitment and fibrotic encapsulation but also leaves adhesive ligands accessible to bacteria, highlighting how tissue regeneration at the biomaterial interface is fundamentally governed by the quality and dynamics of early host cell adhesion. Optimizing these early events represents a key strategy to shift the balance from bacteria colonization toward predictable and stable tissue regeneration, ultimately improving long-term clinical outcomes.

### 2.2 Bacteria adhesion

Following injury or implantation, tissue disruption exposes extracellular matrix (ECM) components, including fibronectin, fibrinogen, and collagen on the wound surface. These exposed matrix proteins provide an adhesive interface that bacteria can exploit to initiate colonization. In particular, β1-integrin–mediated interactions play a central role in linking ECM remodeling at the wound site with bacterial attachment and invasion (Theocharis et al., 2016). In *Staphylococcus aureus*, fibronectin-binding proteins act as molecular bridge between soluble fibronectin and β1-integrins on host cells. This interaction activates α5β1-integrin signaling, induces cytoskeletal rearrangement, and promotes bacterial internalization. Thus, *S. aureus* adhesion is not merely an adhesive event, but an active invasive process that can generate intracellular reservoirs, where pathogens are protected from antibiotics and immune clearance (Kintarak et al., 2004; Edwards et al., 2011). Recent evidence indicates that *S. aureus* uptake depends on β1-integrin recycling and Src/Rac1/PAK1 signaling (Tricou et al., 2024). Moreover, membrane plasticity enables alternative invasion pathways, including αvβ3/αvβ5 integrin-mediated entry, when canonical routes are disrupted (Tricou et al., 2024). Additional mechanisms such as cholesterol-dependent lipid raft-mediated entry and follicular invasion further support the establishment of deep, immune-shielded niches that may reseed infection (Mittal et al., 2019; Nakamura et al., 2021). In *Pseudomonas aeruginosa,* early colonization is driven by type IV pili–mediated adhesion to ECM components, followed by microcolony formation (Pepe et al., 2022; Sánchez-Peña et al., 2024). Retraction of type IV pili also functions as a mechanosensory cue, activating virulence pathways and promoting the transition from planktonic to surface-adapted states (Pepe et al., 2022). Relevantly, *in vivo* transcriptomics revealed a coordinated shift from motility and invasion toward matrix production, quorum sensing, and metabolic adaptation, reflecting progressive tissue colonization (D’Arpa et al., 2021). As infection matures, *P. aeruginosa* forms dense aggregates with activation of denitrification and oxidative-stress pathways, indicating adaptation to hypoxic, nutrient-limited microenvironments (D’Arpa et al., 2021). Importantly, imaging of human chronic wounds demonstrates that *P. aeruginosa* rarely forms surface biofilms but instead occupies subsurface, alginate-encased microcolonies embedded within the wound bed, often surrounded by neutrophil debris and degraded ECM (Kirketerp-Møller et al., 2008; Lichtenberg et al., 2024). These spatially restricted niches create diffusion-limited microenvironments that simultaneously restrict antimicrobial penetration and reinforce metabolic adaptation, thereby promoting persistence (James et al., 2016). In *Streptococcus pyogenes*, colonization relies on high-affinity adhesins and related fibronectin-binding proteins, which tether bacteria to host tissues and actively induce internalization (Molinari et al., 1997; Rohde and Cleary, 2022). Additional surface proteins such as the collagen-like protein Scl1 bind cellular fibronectin, strengthening host-matrix interactions and facilitating spread through tissue layers (Oliver-Kozup et al., 2013). Once within host tissue, *S. pyogenes* secretes effectors including streptolysin O and streptokinase that disrupt immune containment and degrade matrix barriers, while hyaluronidases enable rapid dissemination through soft tissue planes rather than localized colonization (Starr and Engleberg, 2006; O’Seaghdha and Wessels, 2013; Huish et al., 2017). By rapidly anchoring to ECM ligands or invading host cells via alternative routes, pathogens secure a foothold that enables invasion, persistence, and the establishment of infection before effective host regeneration can occur.

### 2.3 Biofilm formation

Biofilm formation can be viewed as the consequence of losing the early “race for the surface.” When host cells fail to rapidly occupy the biomaterial interface or clear the wound infection, attached bacteria can progress into matrix-embedded communities that resist both antibiotics and immune clearance (Figure 1). Once established, biofilms also hinder host-cell attachment and sustain inflammatory signaling, thereby preventing constructive tissue remodeling. On implanted materials, this represents the transition from transient contamination to persistent infection, where regeneration and microbial persistence become incompatible outcomes (Liu et al., 2024). Biofilms are not homogeneous layers of bacterial cells but spatially organized, three-dimensional communities containing microcolonies, water-filled channels and physiologically distinct cellular subpopulations. Their architecture is maintained by a hydrated extracellular polymeric substance (EPS) matrix composed principally of polysaccharides, proteins, extracellular DNA and lipids, although its composition varies according to the microbial strain, implant environment and stage of biofilm maturation (Flemming and Wingender, 2010). In staphylococcal biofilms, important matrix components include polysaccharide intercellular adhesin (PIA), surface and secreted proteins, teichoic acids and eDNA. These components mediate adhesion to the implant, promote cell–cell cohesion and give the biofilm mechanical stability (Flemming and Wingender, 2010). The matrix is not merely a passive structural barrier; barrier; rather it acts as a shared virulence determinant, although its composition and biological effects vary across microorganisms. The matrix generally retains water, nutrients, extracellular enzymes and signalling molecules, supports the establishment of chemical and oxygen gradients, and provides a scaffold within which microbial cells can adopt distinct metabolic states (Flemming and Wingender, 2010). In *S. aureus*, the size and density of an intact biofilm can limit macrophage penetration and phagocytosis, while biofilm-associated signalling and secreted factors attenuate microbicidal inflammatory responses (Thurlow et al., 2011; Gheitasi et al., 2025). Matrix-mediated immune evasion is also observed in other implant-associated pathogens. In *S*. *epidermidis*, PIA is a major determinant of biofilm formation on medical devices. PIA can aid *Staphylococcus epidermidis* evade immune clearance by increasing bacterial survival in human blood and altering complement C5a activity, an effect also associated with clinical prosthetic joint infection (Al-Ishaq et al., 2015). In *P. aeruginosa*, the matrix polysaccharide Psl limits complement deposition, thereby reducing neutrophil and macrophage phagocytosis, oxidative responses and bacterial killing (Mishra et al., 2012). Matrix organization also differs substantially between species; in early *Enterococcus faecalis* biofilms, extracellular DNA localizes to intercellular filamentous structures and to thick layers of extruded extracellular matrix, where it contributes to biofilm cohesion and architectural stability (Barnes et al., 2012). Thus, the matrix can promote microbial virulence through several organism-dependent mechanisms, including mechanical stabilization, immune evasion, metabolic adaptation and protection from environmental stress. It can also support dispersal, allowing individual cells or biofilm aggregates released from the implant surface to colonize surrounding tissues or establish infection at distant sites (Liu et al., 2024). For example, in a polypropylene-mesh infection model, viable *S. aureus* cells were recovered from the tissue surrounding the implant and from the liver and kidneys, consistent with dissemination of bacteria released from the biofilm (Gil et al., 2014). Similarly, in a vascular-catheter model, *S. aureus* biofilms produced bacteria-containing thromboembolic aggregates that adhered to downstream endothelial cells and disseminated to the heart and lungs in mice (Grønnemose et al., 2017). Within these biofilms, pathogens such as *S. aureus*, *P. aeruginosa*, and *S. pyogens* can also deploy multiple cooperative mechanisms to survive antibiotic therapy in the wound implant-associated environments. Although biofilms are often described as antimicrobial resistant, survival during treatment does not always indicate true genetic resistance. In antimicrobial resistance, bacteria acquire heritable changes that allow them to grow at drug concentrations that would normally inhibit them, typically resulting in an increased minimum inhibitory concentration (MIC). Biofilm-associated survival more commonly reflects tolerance, in which bacteria are killed more slowly without a change in MIC. A small fraction of these tolerant cells may enter in a dormant or slow-growing state and survive antimicrobial exposure; these cells are known as persister cells (Brauner et al., 2016). The EPS matrix contributes substantially to this antimicrobial recalcitrance, although the degree and mechanism of protection depend on the matrix composition, microbial strain and physicochemical properties of the antimicrobial agent. Rather than completely preventing penetration in every biofilm, matrix components may delay diffusion or bind antimicrobial molecules, thereby reducing the concentration of active drug reaching embedded cells (Ciofu et al., 2022; Mirghani et al., 2022). Nevertheless, some antimicrobials can penetrate the matrix without effectively eradicating the biofilm, indicating that restricted penetration is not a universal explanation for treatment failure (Boudjemaa et al., 2016). For example, alginate polysaccharides in *P. aeruginosa* biofilms increase matrix viscosity and sequester aminoglycosides antibiotics (Germoni et al., 2016), while polysaccharide intercellular adhesin production in *S. aureus* thickens the matrix and limits antibiotic access (Dunne et al., 1993; Zimmerli and Sendi, 2019; Nguyen et al., 2020). Other matrix polysaccharides may provide drug-specific protection; for example, in *P. aeruginosa*, Psl provides early protection against several antibiotics, and this protection can extend to neighboring Psl-deficient cells incorporated into the biofilm (Billings et al., 2013).

Antibiotic exclusion from biofilms is not solely physical: cationic antibiotics can bind electrostatically to extracellular DNA, reducing free drug availability. In dual-species *S. aureus*-*P. aeruginosa* wound biofilms, DNase treatment restores aminoglycoside activity by releasing sequestered drug from eDNA-bound fractions (Chiang et al., 2013; Wang P. et al., 2024; Wang Z. et al., 2024). Furthermore, biofilm maturation can reduce antimicrobial susceptibility through adaptive mechanisms, including increased activity of biofilm-associated efflux systems, which decrease intracellular antibiotic accumulation without necessarily producing stable genetic resistance (Li et al., 1995; Zhao et al., 1998; Puja et al., 2020). Relevantly, disrupting the biofilm matrix has been shown to improve antimicrobial penetration and activity (Baker et al., 2016; Papadopoulou et al., 2023). However, matrix disruption alone may not eradicate the biofilm because embedded bacteria can remain protected by additional mechanisms, including metabolic inactivity, stress responses, efflux-pumps activity and persister-cell formation. Indeed, beyond physical barriers, biofilm-associated bacteria undergo metabolic reprogramming that promotes tolerance. In hypoxic, nutrient-limited environments, reduced membrane potential limits aminoglycosides uptake (Lobritz et al., 2015; Proctor, 2019; Radlinski et al., 2019), while arginine depletion and activation of alternative pathways further enhance bacterial survival (Freiberg et al., 2024).

Experimental measurements in mature *P. aeruginosa* biofilms have confirmed the presence of large oxygen-limited regions containing slow-growing cells with reduced antibiotic susceptibility (Borriello et al., 2004).

These metabolic adaptations are reinforced by oxidative stress responses. Chronically infected environments expose bacteria to sustained reactive oxygen species (ROS) produced by host immune cells, and *in vivo* wound models demonstrate the induction of antioxidant defenses in *P. aeruginosa,* which promote survival under oxidant stress (Elkins et al., 1999; da Cruz Nizer et al., 2023; Kim et al., 2024; Elkins et al., 1999; da Cruz Nizer et al., 2023; Kim et al., 2024). Importantly, these matrix-associated and physiological mechanisms predominantly produce phenotypic antimicrobial tolerance and should not automatically be equated with heritable antimicrobial resistance. In an arthroplasty-material model, *S. aureus* biofilms retained viable cells after exposure to high cefazolin concentrations without a corresponding increase in MIC, demonstrating tolerance rather than acquired resistance (Urish et al., 2016). Furthermore, biofilm-induced tolerance is not uniform: a study of clinical *P. aeruginosa* isolates found marked strain- and antibiotic-dependent variation in the extent of tolerance (Thöming and Häussler, 2022). Biofilm formation thus represents a major tipping point in the race for the surface, where early failures in host coverage or clearance allow bacteria to establish persistent dominance at the interface.

### 2.4 Polymicrobial and cross-kingdom biofilms

Although *S. aureus* is a major cause of implant-associated infection, clinically relevant biofilms may contain multiple interacting microorganisms. In prosthetic joint infection cohorts, approximately 18%–19% of infections have been classified as polymicrobial, and these infections are frequently associated with lower treatment-success rates than monomicrobial infections (Marculescu and Cantey, 2008). Polymicrobial communities may include coagulase-negative staphylococci, enterococci, streptococci, *P. aeruginosa* and *Escherichia coli*, anaerobic organisms, and, less frequently, fungi. Consequently, infection dynamics are determined not only by the properties of individual pathogens but also by cooperative and competitive interactions within the community.

Bacteria-bacteria interactions can modify biofilm structure, metabolism, virulence, and antimicrobial tolerance. For example, *S. aureus* and *P. aeruginosa* may occupy spatially distinct niches while influencing one another through secreted metabolites, quorum-sensing signals, matrix components, and nutrient competition. In wound-like environments, their coexistence can increase bacterial persistence and antimicrobial tolerance compared with single-species cultures (DeLeon et al., 2014). Conversely, *P. aeruginosa* products can inhibit *S. aureus* respiration and select slow-growing small-colony variants, which are difficult to detect and display enhanced persistence and aminoglycoside tolerance (Vestweber et al., 2024). Thus, apparently antagonistic interactions may nevertheless favor chronic infection by promoting phenotypic adaptation and survival.

Cross-kingdom interactions in clinically-relevant biofilm infections are particularly relevant when bacterial pathogens coexist with fungi such as *C. albicans*. *S. aureus* can adhere to *Candida albicans* hyphae, in part through the fungal adhesin Als3, allowing the fungal network to function as a scaffold for bacterial colonization (Peters et al., 2012). Moreover, β-1,3-glucan and other components of the *C. albicans* extracellular matrix can restrict antimicrobial penetration and protect embedded *S. aureus* (Kong et al., 2016). In a murine catheter model, the presence of *C. albicans* increased *S. aureus* extracellular-matrix production and rendered vancomycin ineffective against the bacterial component of the mixed biofilm (Vila et al., 2021). Cross-kingdom communities can therefore alter biofilm architecture, antimicrobial susceptibility, microbial dissemination, and host inflammatory responses in ways that cannot be predicted from either organism grown alone.

These observations have important implications for multifunctional biomaterial design. Materials that are effective against a single planktonic species or monospecies biofilm may not retain the same activity against a mixed microbial community, particularly when one organism provides structural or metabolic protection to another. Evaluation of anti-infective biomaterials should therefore incorporate clinically relevant multispecies and, where appropriate, bacterial–fungal biofilm models. Such models should assess changes in community composition, spatial organization, matrix production, antimicrobial tolerance, and host-cell responses, in addition to total microbial viability. Biomaterials intended for these settings may require broad-spectrum or combinatorial activity, matrix-disrupting functions, and mechanisms capable of simultaneously supporting immune-mediated clearance and tissue integration (Godoy-Gallardo et al., 2015).

### 2.5 Host cells immune dysregulation

The host immune response plays a central role in determining whether bacterial contamination is successfully cleared or progresses toward persistent infection. Following tissue injury or biomaterial implantation, innate immune cells, particularly neutrophils and macrophages, are rapidly recruited to the affected site, where they contribute to pathogen recognition and clearance through phagocytosis, antimicrobial mediator production, and inflammatory signaling (Biglari et al., 2025; Sweet et al., 2025). Recognition of pathogen-associated molecular patterns by innate immune receptors activates downstream inflammatory pathways and promotes the release of cytokines and chemokines that coordinate additional immune-cell recruitment. Neutrophils act as early effector cells through phagocytosis, degranulation, production of reactive oxygen species, and formation of neutrophil extracellular traps, whereas macrophages contribute both to microbial clearance and to orchestration of the local inflammatory response through cytokine secretion and removal of cellular debris.

These early events form part of the inflammatory phase of physiological tissue healing, which is followed by overlapping proliferative and remodeling phases that are likewise closely regulated by immune cells. In effective tissue regeneration, macrophages transition from early inflammatory (M1) states, characterized by strong cytokine output and microbicidal activity, to later reparative (M2) phenotypes that produce mediators such as TGF-β and IL-10, promoting tissue remodeling (Porcheray et al., 2005; Oneissi Martinez et al., 2008; Mirza et al., 2013; Kimball et al., 2019). M2 macrophages support angiogenesis, fibroblast activity, extracellular matrix deposition, and re-epithelialization through mediators such as TGF-β and VEGF. Adaptive immune cells also contribute to this transition, with T-cell helping regulate inflammatory intensity and resolution and supporting the shift toward tissue repair. During later stages, macrophages and T cells remain involved in controlling inflammation and coordinating extracellular matrix remodeling, highlighting that successful healing depends on a tightly regulated sequence of immune activation, resolution, and regeneration.

Thus, the timing and balance of immune responses are critical for both infection control and tissue regeneration. Persistent bacterial colonization and biofilm formation can disrupt this coordination by impairing immune-cell function and sustaining or redirecting inflammatory responses, ultimately promoting chronic inflammation, defective tissue repair, and a microenvironment that favors bacterial persistence and tissue damage (Biglari et al., 2025; Brandquist and Kielian, 2025; Sahu and Ruhal, 2025; Sweet et al., 2025). Biofilm-forming bacteria actively contribute to this immune imbalance. Notably, *S. aureus* biofilms dramatically suppress macrophage inflammatory pathways, reducing IL-1β, TNF-α and inducible nitric oxide synthase (iNOS) production (Thurlow et al., 2011), while inducing arginase-1 to divert L-arginine metabolism away from bactericidal nitric oxide production toward pro-fibrotic repair, facilitating immune evasion (Thurlow et al., 2011). They also reshape adaptive immunity: biofilm-produced α-toxin has been shown to skew responses toward sustaining neutrophil-driven inflammation (Bonifacius et al., 2020). A prosthetic-joint infection model combined with a transposon screen identified bacterial lactate biosynthesis as a key driver of immune dysregulation, with biofilm-derived lactate enhancing IL-10 production in macrophages and promoting the stabilization of an immunosuppressive, pro-biofilm compartment (Brandquist and Kielian, 2025). This establishes a feedback loop in which bacterial metabolism directly stabilizes an immunosuppressive myeloid compartment that favors biofilm persistence. Chronic *P. aeruginosa* infection actively dampens innate immune function through coordinated toxin secretion, metabolic manipulation, and biofilm-driven reprogramming. Bacteria-produced rhamnolipids cause rapid neutrophil necrosis, loss of membrane integrity, and release of cytotoxic granule enzymes, thereby preventing effective phagocytosis and amplifying tissue damage (Jensen et al., 2007; Alhede et al., 2009; Soberón-Chávez et al., 2021). The pigment pyocyanin penetrates neutrophil mitochondria, triggering a burst of mitochondrial ROS production, activation of acid sphingomyelinase, ceramide production, cytochrome c release and apoptosis (Rada et al., 2013). In primary human neutrophils, pyocyanin also enhances neutrophil extracellular trap formation via NADPH oxidase–dependent extracellular superoxide production, shifting ROS from intracellular microbicidal bursts toward extracellular DNA–protease networks that can damage host tissue and enhance chronic inflammation (Rada et al., 2013). Complementing these neutrophil-targeted mechanisms, *P. aeruginosa* also deploys outer-membrane vesicles that directly reprogram macrophage signaling, effectively steering macrophages into metabolically rewired, dysfunctional phenotypes that support chronic infection (Li et al., 2025). Biofilm metabolism provides an additional axis of immune dysregulation. *P. aeruginosa* biofilms sustain neutrophil influx while suppressing their gene expression and altering macrophage recruitment, producing a non-resolving inflammatory infiltrate that delays infection healing (Trøstrup et al., 2017). By continuously reprogramming host immune responses toward dysfunctional, non-resolving states, bacteria maintain dominance at the biomaterial interface, suppress clearance mechanisms, and stabilize a niche that favors their long-term persistence and their advantage in the race for the surface.

### 2.6 Microenvironmental disruption

Beyond immune dysregulation, bacterial infection profoundly alters the physicochemical landscape of the wound or of the implant-surrounding environment, creating a self-reinforcing landscape that impairs repair while sustaining microbial persistence. These changes do not occur in isolation; rather, they interact to establish a hostile microenvironment that disrupts the coordinated cellular processes required for healing (Nauta et al., 2014; Rembe et al., 2026). A defining feature of chronically infected environments is spatial and temporal heterogeneity in oxygen availability. Poor perfusion, microvascular damage, and edema limit oxygen delivery, while infiltrating immune cells and proliferating bacteria increase metabolic demand, leading to steep oxygen gradients and regions of sustained hypoxia (André et al., 2022). Hypoxia stabilizes hypoxia-inducible factor 1-alpha (HIF-1α), which transiently promotes adaptive responses such as angiogenesis through upregulation of vascular endothelial growth factor (VEGF). However, under persistent hypoxic conditions, sustained HIF-1α activation becomes maladaptive, leading to impaired vascularization, metabolic dysfunction, and reduced fibroblast-mediated matrix production (Taylor and Colgan, 2017). Fibroblasts exposed to prolonged hypoxia exhibit reduced proliferation and impaired collagen synthesis, directly compromising granulation tissue formation (Han et al., 2023). In parallel, endothelial cell dysfunction limits neovascularization, preventing restoration of adequate perfusion and thereby maintaining the tissue in a hypoxic state (Han et al., 2023). Infection is also associated with persistent redox imbalance, driven by excessive production of ROS from activated neutrophils and macrophages (Hunt et al., 2024). While ROS such as superoxide and hydrogen peroxide are essential for antimicrobial defense, their sustained overproduction overwhelms endogenous antioxidant systems, leading to oxidative stress (Wang et al., 2023; Hunt et al., 2024). This damages lipids, proteins, and DNA, disrupts mitochondrial function, and induces cellular senescence or apoptosis in keratinocytes and fibroblasts. Importantly, oxidative stress also alters signaling pathways involved in migration and proliferation, further delaying re-epithelialization (Wang et al., 2023; Hunt et al., 2024). Another critical but often underappreciated factor is pH dysregulation (Rembe et al., 2026). In acute wounds, including those created during implant surgery, the wound microenvironment often shifts towards a more alkaline pH (Sim et al., 2022; Rembe et al., 2026). These alkaline conditions are conducive to bacterial proliferation, enhance bacterial virulence and biofilm stability while simultaneously reducing oxygen release from hemoglobin via the Bohr effect, thereby exacerbating local hypoxia. In addition, persistent inflammation drives elevated secretion of proteolytic enzymes, particularly matrix metalloproteinases, alongside neutrophil elastase and other degradative factors, further destabilizing tissue architecture (Percival et al., 2014). This prevents the formation of a stable scaffold required for cell migration, cell adhesion, and tissue organization, effectively halting progression from the inflammatory to the proliferative phase of healing (Jiang et al., 2025). Collectively, these alterations generate a dysfunctional microenvironment characterized by hypoxia, oxidative stress, altered pH, and uncontrolled ECM degradation. This environment not only impairs the cellular and molecular programs required for tissue regeneration, but also reinforces bacterial survival and persistence, creating a vicious cycle that prevents effective healing and shifts the balance in the race for surface colonization.

## 3 Design strategies in modern multifunctional biomaterials

The intertwined mechanisms orchestrating tissue regeneration demonstrate the need for biomaterials that act beyond conventional antimicrobial protection. The following section examines major multifunctional design strategies that combine microbial control with immune regulation, microenvironmental restoration, and tissue regeneration.

### 3.1 Metal ion-based biomaterials

The direct incorporation and controlled release of antibiotics from biomaterials have been widely explored to reduce bacterial adhesion and inhibit biofilm formation (Wang et al., 2016; Tavoni et al., 2025; Wang et al., 2016; Tavoni et al., 2025). However, the rapid rise of multidrug-resistant pathogens has not only highlighted the limitations of conventional antibiotic-based approaches, but also created an opportunity to explore alternative strategies (Habib et al., 2025; Habib et al., 2025). In this context, ion-based approaches have emerged as promising multifunctional platforms for combined antimicrobial and regenerative applications. Metal ions-based materials release biologically active ions (e.g., Ag^+^, Zn^2+^, Ca^2+^) upon degradation, which exert broad-spectrum antimicrobial effects (Elahpour et al., 2024). Indeed, positively charged ions interact electrostatically with negatively charged bacterial membranes, increasing membrane permeability and leading to leakage of intracellular components (Lemire et al., 2013). In addition, these ions can penetrate the cell envelope and bind intracellular targets, including proteins and nucleic acids, thereby disrupting enzymatic activity and inhibiting DNA replication (Godoy-Gallardo et al., 2021; Xu Z. et al., 2021). More recently, their antibacterial activity has been strongly linked to the ROS generation, often via Fenton-like reactions (Ng et al., 2011; Ning et al., 2015), ultimately contributing to cell death (Ning et al., 2015; Godoy-Gallardo et al., 2021). These mechanisms have provided further insights into ion-mediated antibacterial activity and have guided the design of more effective antimicrobial systems. From a biological perspective, these ions also play a key role in modulating the host microenvironment and supporting tissue regeneration. Mg^2+^ and Ca^2+^ ions are known to promote angiogenesis by stimulating endothelial cell proliferation and migration, as well as enhancing the release or intracellular signalling of pro-angiogenic factors such as VEGF (Moccia et al., 2019; Manescu et al., 2023). Mg^2+^ and Ca^2+^ ions play a central role in bone regeneration: Mg^2+^ through the activation of Wnt/β-catenin, MAPK/ERK, and PI3K/Akt signaling pathways (Chen Y. et al., 2024) and Ca^2+^ by acting as a secondary messengers that activate calcium-dependent signaling pathways (e.g., calmodulin/CaMK and Wnt/β-catenin), ultimately enhancing bone formation (Hu et al., 2024; Tan et al., 2025). Phosphate ions (PO_4_^3−^), by contributing to mineral nucleation and hydroxyapatite formation, further support matrix mineralization (Michigami and Ozono, 2019). Similarly, Zn^2+^, acting as a cofactor for numerous enzymes, including matrix metalloproteinases (MMPs) and DNA/RNA polymerase, is involved in ECM remodeling and cell proliferation (Nosrati et al., 2019; Nosrati et al., 2019). It also influences cell signaling pathways (e.g., NF-κB and MAPK), contributing to controlled inflammatory responses and immune modulation (Gammoh and Rink, 2017). Additionally, Zn^2+^ has been shown to support osteogenesis by enhancing alkaline phosphatase (ALP) activity and collagen synthesis, while maintaining a balance between inflammation and tissue regeneration (Seo et al., 2010).

In this scenario, it is clear that the incorporation of therapeutic ions represents a powerful strategy for developing multifunctional biomaterials capable of simultaneously addressing infection and tissue repair, as summarized in Table 1.

**TABLE 1:** Ion-releasing biomaterials with combined antimicrobial and tissue-regenerative activity.

References	Type of material and ion released	Effect on bacteria	Effect on tissue regeneration
Zheng et al. (2026)	Sodium alginate/glycyrrhizic acid hydrogel releasing Cu^2+^, Zn^2+^, Ca^2+^	Broad-spectrum antibacterial activity against *S. aureus* and *E. coli*	Promoted keratinocyte/fibroblast growth and migration, angiogenesis, collagen deposition, M2 macrophage polarization, and wound closure
Liu Y. et al. (2025)	Schiff-base HA/alginate nanocomposite hydrogel with Cu/Ag nanoparticles releasing Cu^+^/Cu^2+^ and Ag^+^	Chemodynamic antibacterial activity against *E. coli* and *S. aureus*; Ag^+^ acted synergistically with Cu-mediated ROS generation	Promoted angiogenesis/tube formation *in vitro*; enhanced wound repair *in vivo*
Zhang et al. (2025)	GelMA@Sr-ZIF-8 hydrogel releasing Zn^2+^ and Sr^2+^	Antibacterial activity against *S. aureus* and *E. coli*; inhibited biofilm formation	Reduced inflammation, enhanced macrophage phagocytosis, promoted osteoblast proliferation/differentiation, inhibited osteoclast activity, and enhanced angiogenesis
Luo et al. (2025)	Piezoelectric PLLA fibers containing Cu-doped bioactive glass; release of Cu^2+^	Strong activity against MRSA, enhanced by ultrasound/piezoelectric stimulation	Promoted antibacterial-immune regulation, macrophage activation, and wound healing
Thongnuek et al. (2025)	Ag-doped core–shell mesoporous bioactive glass nanoparticles releasing Ag^+^ and bioactive glass dissolution ions	Antibacterial activity against MRSA and *E. coli*	Supported hMSC viability, osteogenic differentiation, and mineralization
Sánchez-Salcedo et al. (2023)	3D-printed Ag-containing mesoporous bioactive glass scaffolds releasing Ag^+^ and glass-derived ions	Antibacterial and antibiofilm activity	Supported MC3T3-E1 preosteoblast viability and normal morphology
El-Fiqi et al. (2021)	Cu-doped mesoporous bioactive glass nanospheres loaded with EGF; release of Cu^2+^, Ca^2+^, silicate ions	Antibacterial activity against *Enterococcus faecalis*, relevant to infected dental pulp models	Enhanced HUVEC migration, tube formation, VEGF expression, and osteo/odontogenic differentiation of human dental pulp stem cells; promoted regeneration in an infected rat molar defect model
Chen et al. (2022)	Zinc-based MOF, Zn-BTC, with slow release of Zn^2+^	Bactericidal effect against drug-resistant bacteria, including reduced MRSA and *E. coli* viability	Promoted fibroblast proliferation and migration, reduced oxidative/inflammatory markers, increased wound-healing genes such as TGF-β and type I collagen, and improved rat skin wound healing
Wang S. et al. (2024)	Injectable chitosan/hyaluronic acid dual-network hydrogel containing Cu/Sr-doped mesoporous bioactive glass and BMP-2; release of Cu^2+^ and Sr^2+^	Antimicrobial activity against *E. coli* and *S. aureus*	Combined anti-inflammatory, pro-angiogenic, and bone-regenerative effects for infected bone defect healing
Zhang X. et al. (2023)	Li/Co-doped mesoporous bioactive glass nanoparticles releasing Li^+^ and/or Co^2+^	Antimicrobial activity against *E. coli* and *S. aureus*	Stimulated osteogenesis and angiogenesis, supporting potential use as bioactive fillers for bone repair

In a recent work, Cu^2+^, Zn^2+^ and Ca^2+^ were incorporated into a sodium alginate matrix to leverage distinct therapeutic effects: Cu^2+^ ions provide antibacterial activity and promote angiogenesis; Zn^2+^ synergistically enhance antibacterial efficacy while offering antioxidant protection; and Ca^2+^ facilitate structural cross-linking and support cell migration. Glycyrrhizic acid further added anti-inflammatory activity and improved hydrogel mechanics (CZC-SA-GA hydrogel) (Figure 2A). The resulting composite hydrogel showed broad-spectrum antibacterial activity against *S. aureus* and *Escherichia coli*, supported keratinocyte and fibroblast growth and migration, promoted angiogenesis through enhanced tube formation and VEGF expression, and reduced macrophage inflammation by decreasing NO production, downregulating IL-6 and iNOS, and promoting M2 polarization. *In vivo*, the hydrogel accelerated full-thickness wound healing, with improved closure, reduced inflammation, increased collagen deposition, and enhanced neovascularization (Figure 2B) (Zheng et al., 2026). A key aspect of ion-releasing systems lies in the ability to precisely control degradation kinetics, thereby enabling sustained and localized release. By tailoring material composition, it becomes possible to maintain therapeutic ion concentrations over time, ensuring prolonged antimicrobial efficacy while continuously supporting cellular processes. In particular, the incorporation of pH-responsive components allows materials to undergo accelerated degradation under acidic conditions, such as those found in infected or inflamed tissues, thereby enabling on-demand ion release at the pathological site while limiting premature release in healthy environments. For instance, Liu Y. et al. (2025), incorporated cuprous–benzoylthiourea-modified silver nanoparticles within a Schiff base-crosslinked hyaluronic acid-sodium alginate hydrogel. Schiff base bonds are acid-labile and under simulated infected conditions (acidic pH with hyaluronidase), the composite hydrogel exhibited gradual degradation, with over 80% mass loss by day 5 and complete degradation by day 10. This degradation enabled a sustained release of ion nanoparticles, which retained their structural integrity and functional activity, underscoring the efficacy of these gels for both immediate antibacterial action and prolonged therapeutic effects through controlled degradation and ion/nanoparticle release. Indeed, at the site of infection, the nanocomposite system enables Cu^+^-mediated chemodynamic (ROS generation) antibacterial action against *E. coli* and *S. aureus*, while Ag^+^ ions contribute synergistically to bacterial eradication. Here, the presence of benzoylthiourea was used to facilitate the continuous reduction of Cu^2+^ to Cu^+^, thereby sustaining and enhancing chemodynamic antibacterial therapy. This synergistic mechanism allows for effective antibacterial activity even at relatively low silver concentrations, lowering the risk of silver-induced toxicity and highlighting a critical therapeutic window. Finally, the combined action of copper and hyaluronic acid promoted angiogenesis, as evidenced by enhanced tube formation with more organized vascular-like structures, highlighting its potential as an advanced therapeutic strategy for infected wound management (Liu Y. et al., 2025). Recently, metal–organic frameworks have gained a lot of attention for their pH-responsive release mechanism. These are a class of crystalline porous materials composed of metal ions coordinated with organic ligands, widely recognized for their capacity to store and release therapeutic ions in a controlled manner (Zhao C. et al., 2023; Zhao Y. et al., 2024). Indeed, unlike conventional ion-doped materials that release ions passively, metal–organic frameworks introduce a pH-responsive release mechanism. They remain relatively stable under physiological conditions but degrade faster in acidic, pathological environments, ensuring that therapeutic ions are released on-demand at infected or diabetic inflamed sites (Zhang et al., 2025). In this context, Zhang et al. (2025) incorporated Sr ions into Zn-containing organic frameworks (zeolitic imidazolate framework-8, ZIF-8), which was subsequently embedded within a (gelatin methacryloyl) GelMA matrix to fabricate a multifunctional hydrogel tailored to the pathological microenvironment of diabetic acidic tissues (GelMA@Sr-ZIF-8 hydrogel). The pH-responsive degradation, leading to enhanced release of Zn^2+^ and Sr^2+^ ions, provided strong antibacterial activity against *S. aureus* and *E. coli* by directly killing planktonic bacteria and inhibiting biofilm formation. Meanwhile, Zn^2+^ and Sr^2+^ ions synergistically contributed to reverse the pro-inflammatory microenvironment, and restoring macrophage function to enhance phagocytosis and eliminate residual bacteria within mature biofilms. In addition, Sr^2+^ ions stimulated osteoblast proliferation and differentiation, inhibiting osteoclast activity, and enhancing angiogenesis at defect sites (Zhang et al., 2025). Another representative MOF-based ion-releasing system was reported by Chen et al. (2022), who developed a nanosized zinc-based metal–organic framework, Zn-BTC, by coordinating Zn^2+^ with trimesic acid (H_3_BTC) (Chen et al., 2022). The resulting MOF displayed a porous, stable framework with nanoscale rod-like morphology and was designed to release Zn^2+^ gradually under physiological conditions (Figure 2C), while undergoing faster degradation in a weakly acidic environment. *In vitro*, Zn-BTC exhibited antibacterial activity against multidrug-resistant pathogens, reducing MRSA and *E. coli* viability by approximately 41.4% and 47.2%, respectively (Figure 2D) and accelerated wound closure *in vivo* (Figure 2E), highlighting its potential as a multifunctional MOF platform that combines Zn^2+^-mediated antibacterial, anti-inflammatory, and pro-regenerative effects (Chen et al., 2022). Within the broader framework of ion-releasing systems for antimicrobial and regenerative applications, bioactive glasses (BGs) have emerged as particularly attractive candidates. Their ability to degrade in physiological environments and release biologically active ions in a controlled manner makes them ideal platforms for simultaneously modulating bacterial activity and promoting tissue regeneration. In this context, BGs are a class of surface-reactive glass-ceramic biomaterials, that degrade upon contact with physiological fluids (Nandi et al., 2016; Madival and Rajiv, 2025; Zhu Y. et al., 2025).

**FIGURE 2:**
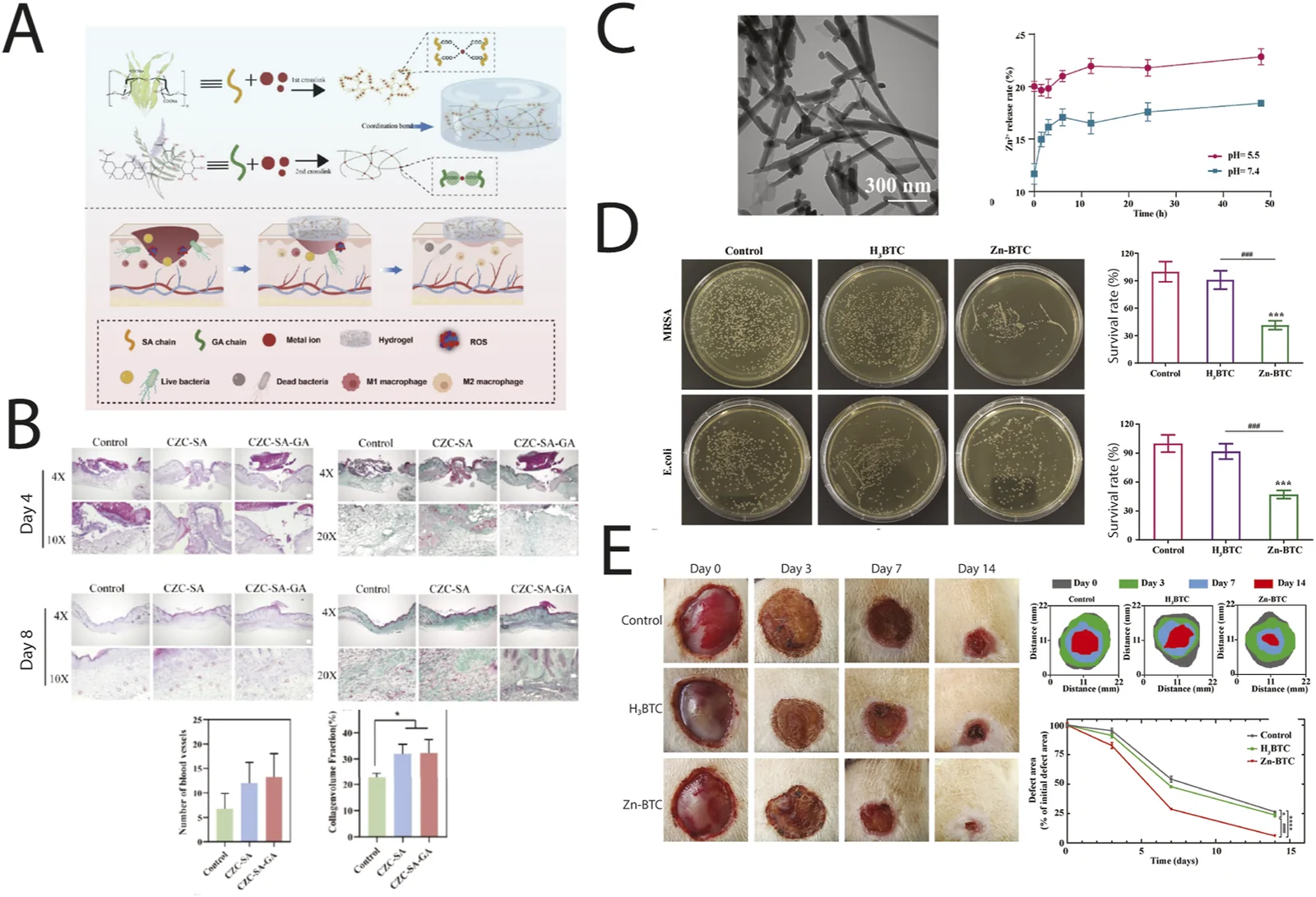
Metal-based platforms for infected wound healing and bone regeneration. **(A)** Schematic illustration of CZC-SA-GA hydrogel formation and its proposed mechanism for promoting infected wound healing through antibacterial activity, ROS regulation, macrophage polarization, angiogenesis, and tissue repair. **(B)** H&E and Masson’s trichrome staining of wound tissues after 4 and 8 days of treatment with different hydrogels. The corresponding quantitative analyses show the number of newly formed blood vessels and the relative collagen content at the wound site. Adapted from (Zheng et al., 2026) with permission from American Chemical Society. **(C)** On the left, TEM micrographs showing the rod-like morphology of Zn-BTC. On the right, Zn^2+^release rate in physiological and acidic conditions. **(D)** Antibacterial activity of Zn-BTC against MRSA and *E. coli*. **(E)** Representative images of *in vivo* experiments with wound area treated with different materials, and their corresponding schematic image with line graphs showing wound area changes versus time. Adapted from (Chen et al., 2022) with permission from Oxford University Press.

Common network-forming oxides include SiO_2_, B_2_O_3_, and P_2_O_5_, giving rise to silicate-, borosilicate-, borate-, and phosphate-based glasses (Madival and Rajiv, 2025; Zhu Y. et al., 2025). As a result of this composition and reactivity, BGs possess inherent antimicrobial effects primarily attributed to local pH elevation, osmotic stress resulting from glass dissolution (Raimondi et al., 2022; Elahpour et al., 2024). Importantly, beyond their antibacterial action, BGs are also inherently bioactive, actively participating in regenerative processes (Pajares-Chamorro and Chatzistavrou, 2020; Vallet-Regi and Salinas, 2021). These intrinsic properties are further amplified by BGs doping, which can be introduced during synthesis and allow for a homogeneous distribution of ions throughout the glass network (Godoy-Gallardo et al., 2021). The incorporation of metal ions into the BG network further amplifies their antibacterial effect. For example, silver-doped BGs exhibit strong bacteriostatic and bactericidal activity against pathogens like *Escherichia coli*, *P. aeruginosa*, and *S. aureus*, with near-complete bacterial elimination observed within 24 h depending on doping concentration (Ahmed et al., 2006; Elahpour et al., 2024). Similarly, copper-doped BGs demonstrate dose-dependent antibacterial effects, including inhibition of biofilm formation and bacterial viability, although typically at higher concentrations than silver (Fakher and Westenberg, 2024). Beyond antimicrobial activity, metal-ion doped BG nanoparticles have also been used in tissue regeneration (Pajares-Chamorro and Chatzistavrou, 2020; Prasad et al., 2026), thereby enabling their use as dual-function biomaterials (Prasad et al., 2026). For instance, the incorporation of silver ions into BGs nanoparticles obtained with a sol-gel approach was used to endow the nanosystems with antibacterial activity against *S. aureus* and *E. coli.* Notably, in addition to this antibacterial effect, the doped BGs nanoparticles demonstrated strong osteoconductive potential in *in vitro* bioactivity studies, indicated by the rapid formation of a hydroxyapatite layer, and supported growth and proliferation of osteoblastic-like cells (Prasad et al., 2026). Among BGs, mesoporous BGs (MBGs), with ordered pore structures in the mesoporous range (5–20 nm), have gained a lot of attention for their increased surface area and excellent potential for therapeutic ions release (Elahpour et al., 2024). In particular, Ag-doped core–shell mesoporous BGs have been synthesized using a modified sol–gel approach and showed significantly higher surface area (approximately 860 m^2^/g) compared to solid particles (around 17 m^2^/g), along with substantially increased pore volume. Relevantly, the core-shell mesoporous architecture granted sustained and controlled release for over 21 days at sub-microgram-per-milliliter concentrations. As a result, Ag-doped mesoporous BGs promoted faster mineralization, exhibited potent antibacterial effects against methicillin-resistant *S. aureus* (MRSA) and *E. coli*, and supported both viability and osteogenic differentiation of human mesenchymal stem cells, highlighting how tailoring nanostructure enables precise control over ion release and biological response, advancing BGs as multifunctional materials for regenerative and antimicrobial applications (Thongnuek et al., 2025). In addition, ion-doped BGs have been successfully incorporated into composite systems such as scaffolds (Sánchez-Salcedo et al., 2023), polymers (Wang et al., 2019), and coatings (Li et al., 2016; Ao et al., 2025) to combine antibacterial functionality with tissue regeneration. For instance, silver-doped MBGs have been integrated into 3D-printed scaffolds for bone regeneration. Sánchez-Salcedo et al. (2023) demonstrated that Ag-containing MBG scaffolds fabricated via rapid prototyping exhibited strong antibacterial activity and biofilm inhibition, while supporting MC3T3-E1 preosteoblastic cell viability and maintaining normal cell morphology. Similarly, copper-doped BG nanoparticles have also been embedded in polymer matrices such as polycaprolactone (PCL), improving both antibacterial performance and bioactivity (Wang et al., 2019). For example, Wang et al. (2019) incorporated Cu-containing BG nanoparticles into PCL scaffolds. Compared to pure PCL scaffolds, scaffolds incorporating Cu-doped BGs upregulated early-stage osteogenic markers in mesenchymal cells, while increasing von Willebrand factor (vWF) gene expression in endothelial cells, thus underscoring the enhanced angiogenic effect associated with Cu^2+^ release (Wang et al., 2019). Furthermore, Cu-doped BG coatings showed pro-angiogenic potential, stimulating pathways such as HIF-1α and VEGF, and promoting vascularization in wound healing applications (Li et al., 2016). Moving toward more advanced designs, ion-doped BGs can be incorporated into increasingly complex, multifunctional systems. For example, Cu-doped BGs nanoparticles were incorporated into polarized PLLA fibers, yielding a piezoelectric composite. Under the combined influence of ultrasound and released Cu^2+^ ions, the composite material demonstrated strong antibacterial effects against methicillin-resistant *S*. *aureus*, while promoting polarization of macrophages toward the M1 phenotype, enhancing their phagocytic and bactericidal activity both *in vitro* and *in vivo* (Luo et al., 2025). Overall, the continuous evolution of ion-doped BG systems, from simple particles to structurally and functionally complex platforms, highlights their adaptability in addressing both infection control and tissue repair. This versatility, combined with tunable composition and architecture, positions BG-based materials as promising candidates for next-generation therapeutic strategies.

Despite their considerable potential, the translational maturity of metal- and ion-based biomaterials remains highly variable. Silver-based antimicrobial dressings and coatings represent some of the most clinically established examples, and silver-containing wound dressings have been evaluated in human studies, including randomized clinical trials and meta-analyses (Liang et al., 2024; Yi et al., 2024). However, major clinical challenge still hinder metal- and ion-based biomaterials translations, including a relatively narrow therapeutic window of many antimicrobial ions, since concentrations sufficient for bacterial killing may also induce oxidative stress, cytotoxicity, or impaired function in host cells (Wang C. et al., 2025; Zhu C. et al., 2025). Precise control over ion loading, release kinetics, local concentration, degradation, and systemic exposure is therefore essential. Additional concerns include accumulation or persistence of non-degradable metallic components and long-term tissue responses highlighting the need for a careful balance between antimicrobial potency and host compatibility, and material designs that minimize systemic exposure while preserving regenerative efficacy (Wang C. et al., 2025; Zhu C. et al., 2025).

### 3.2 Ionic liquid-based systems

Ionic liquids (ILs) have emerged as a powerful and versatile class of functional materials for antimicrobial and regenerative applications. ILs are organic salts composed of bulky cations and anions that remain liquid at relatively low temperatures, while exhibiting intrinsic ionic conductivity and highly tunable physicochemical properties (Nikfarjam et al., 2021; Sharma et al., 2024). A defining advantage of many IL-based systems is their predominantly contact-active antimicrobial mechanism, which sets them apart from conventional ion- or drug-releasing materials. Rather than relying primarily on diffusion, surface-confined ILs can directly interact with negatively charged bacterial membranes through electrostatic forces, inducing membrane disruption, increased permeability, and ultimately cell lysis (Hu Y. et al., 2023; Sharma et al., 2024). This mechanism can overcome the limitations of burst release and rapid depletion, while potentially reducing concentration-dependent cytotoxicity and enabling persistent, long-term antibacterial activity at the material interface (Hu Y. et al., 2023; Sharma et al., 2024). Importantly, ILs go beyond static antimicrobial function by enabling dynamic ionic interactions with the biological microenvironment, thereby actively modulating cell–material interfaces and biological responses. ILs can interact with eukaryotic cell membranes through more subtle and tunable mechanisms, which are highly dependent on their chemical structure (Fernandes et al., 2022; Hu Y. et al., 2023; Sharma et al., 2024). While the cationic components can associate with the negatively charged phospholipid head groups, their interaction is generally less disruptive than in bacterial membranes, due to the presence of cholesterol and the more complex, zwitterionic nature of mammalian membranes (Gupta et al., 2025a; Gupta et al., 2025b). At controlled concentrations, ILs can modulate membrane fluidity and permeability without inducing lysis, thereby influencing processes such as cell adhesion, ion transport, and intracellular signaling (Kumari et al., 2020; Egorova et al., 2024; Sharma et al., 2025). In particular, IL-induced changes in membrane polarization and ionic flux can activate signaling pathways related to proliferation and differentiation, contributing to tissue regeneration (Kumari et al., 2020; Egorova et al., 2024; Sharma et al., 2025). Notably, these membrane-selective interactions also underpin the emerging use of ILs in anticancer applications. Cancer cell membranes typically exhibit higher negative surface charge, altered lipid composition, and reduced cholesterol content compared to healthy cells, making them more susceptible to IL-induced membrane disruption (Ali et al., 2023; Mohd Noor et al., 2024). As a result, certain ILs can selectively induce cytotoxicity in cancer cells while sparing normal cells, through mechanisms involving membrane permeabilization, mitochondrial dysfunction, and oxidative stress generation (Ali et al., 2023; Mohd Noor et al., 2024). The ability to differentially interact with bacterial and mammalian membranes positions IL-based systems as a distinct and advanced alternative to traditional release-based strategies, capable of integrating sustained antimicrobial performance with adaptive biological activity. This versatility has enabled ILs to be applied across multiple biomedical contexts, particularly in wound-care platforms where antimicrobial and regenerative demands overlap. For example, hydrogels composed of poly (vinyl alcohol), acrylamide, and 1-vinyl-3-butylimidazolium bromide were developed and exhibited concentration-dependent antibacterial activity against bacteria, fungi, and mold, while accelerating wound healing in murine models (Fang et al., 2019). Building on this concept, IL incorporation has also been used to improve mechanical stability and impart self-healing properties. For instance, lignin/polyionic liquid hydrogels with self-healing and mechanically robust properties have been developed. These materials maintain antibacterial efficacy after repeated use and promote accelerated wound healing *in vivo* (Datta et al., 2025). Furthermore, stimuli-responsive IL-based systems enable adaptive wound care; for instance, poly (vinyl alcohol)-based hydrogels with pyrrolidinium ILs and borate crosslinkers, further enable adaptive wound care through pH and glucose responsiveness while retaining strong antibacterial activity (Datta et al., 2025). The multifunctionality of ILs has also been extended from soft wound dressings to mineralized and tissue-regenerative matrices. Imidazolium-based ILs have been incorporated into calcium phosphate bionanocomposites through an *in situ* sol-gel process, generating injectable materials with combined antimicrobial, anti-inflammatory, and osteogenic properties (Raucci et al., 2018). In this system, the IL structure, particularly the alkyl chain length, influenced calcium phosphates crystallization and the resulting biological response. Specifically, the ILs acted as structure-directing additives, affecting the organization and crystallinity of the calcium phosphate phase, while also providing intrinsic antimicrobial activity in the final composite. Biocomposites containing long-chain ILs exhibit effective antimicrobial activity while supporting mesenchymal stem cell proliferation and osteogenic differentiation, demonstrating their potential for infection-resistant bone regeneration (Raucci et al., 2018). To localize and better control ionic functionality and limit burst exposure and potential cytotoxicity, imidazolium poly (ionic liquid) microspheres have also been explored and incorporated into GelMA–PEG hydrogels, enabling sustained antibacterial and anti-inflammatory effects while preserving the tissue-mimetic properties of the GelMA–PEG matrix. In this work, the microspheres acted as the main bioactive component, with imidazolium groups interacting with bacterial membranes and contributing to strong activity against *E. coli* and *S. aureus*. Their incorporation also improved the anti-inflammatory profile of the hydrogel, as shown by reduced expression of inflammatory mediators such as TNF-α, IL-6, and IL-1β. Beyond infection and inflammation control, the microsphere-loaded hydrogels supported human skin fibroblast and endothelial-cell migration and promoted osteogenic differentiation *in vitro*, highlighting their potential for tissue-repair applications (Zhou C. et al., 2024; Zhou M. et al., 2024). Combination strategies that pair ILs with bioactive metal ions have also been investigated to broaden antimicrobial functionality. For example, electrospun nanofibrous membranes incorporating imidazolium-type poly (ionic liquid)s and Ce ions were developed to combine antibacterial activity with resistance-gene degradation capability. These membranes showed antimicrobial efficacy against *E. coli*, kanamycin-resistant *E. coli*, *S. aureus*, and MRSA, while also exhibiting DNase-mimic activity toward the resistance genes k*anR* and mecA. By coupling bacterial eradication with degradation of released antibiotic-resistance genes, these membranes enhanced wound healing and may be employed to reduce the environmental dissemination of antibiotic resistance, supporting their potential as “green” wound dressings for clinical applications (Luo et al., 2021). More complex composite designs have further exploited the integration of ILs with functional nanomaterials to achieve simultaneous infection control and tissue regeneration. In this context, a composite gel has been developed that combines a polyionic liquid hydrogel with ZnO–Au nanoparticles to achieve both strong antibacterial activity and enhanced tissue regeneration. The ionic liquid, integrated into the hydrogel network, provides intrinsic antibacterial effects against *E. coli* and *S. aureus* through electrostatic interactions with bacterial membranes and improves conductivity, enabling efficient transmission of piezoelectric signals under ultrasound stimulation. At the same time, embedded ZnO nanoparticles release Zn^2+^ ions in a controlled manner, promoting endothelial cell proliferation, migration and angiogenesis. Together with ultrasound-triggered ROS generation and electrical stimulation, these components act synergistically to reduce infection, modulate inflammation, and accelerate wound healing, demonstrating a promising strategy for advanced wound care (Ju et al., 2025). ILs have also been added to polymeric substrates to enhance substrate conductivity, demonstrating potential for regeneration strategies in electroactive tissues (Meira et al., 2019; 2022; Carvalho et al., 2024). For instance, poly vinylidene fluoride-trifluoroethylene piezoelectric films incorporating the ionic liquid 1-ethyl-3-methylimidazolium hydrogen sulfate were shown to possess strong antibacterial activity, effectively limiting biofilm of *S. aureus* and *E. coli*. ILs incorporation also enhanced osteogenesis by increasing calcium deposition, upregulating osteogenic genes (e.g., RUNX2, BMP2), and promoting calcium signaling through the upregulation of voltage-gated and mechanosensitive calcium channels (i.e., CACNA2D1 and PIEZO1), highlighting how the electromechanical (piezoelectric and piezoionic) properties of a substrate may be used to direct cellular behavior (Carvalho et al., 2024). Similarly, deep eutectic solvent-based ionic gels have emerged as multifunctional matrices for electrically responsive wound dressings. In one example, a choline chloride/ethylene glycol ionic gel provided ionic conductivity, water-free stability, flexibility, and a suitable platform for incorporating antibacterial Zn^2+^ ions. This stable ionogel dressing supported on-demand electrical stimulation while maintaining antibacterial activity against both *E. coli* and *S. aureus*. Beyond infection control, the system accelerated wound closure and promoted epidermal regeneration, neo-epithelium formation, angiogenesis, and collagen deposition, demonstrating the broader potential of IL-like ionic matrices for regenerative wound care (Yang et al., 2025). The conductivity-enhancing role of ILs is also relevant for bioelectronic and sensing applications, where antimicrobial or regenerative materials can be further integrated with real-time monitoring functions. Indeed, the incorporation of ILs into a cellulose nanofiber hydrogel was used to endow the polymer matrix of hydrogel with high ionic conductivity and excellent strain sensitivity and provide real-time bioelectronic monitoring capabilities, offering an integrated platform for advanced, responsive biomedical applications (Zhu et al., 2023). In this system, ILs-based hydrogels enabled to accurately monitor a wide range of physiological signals, including body movements of varying intensity, frequency, and type, as well as subtle electrophysiological signals such as electromyography and electrocardiogram signals and pulse (Zhu et al., 2023).

### 3.3 Phototherapeutic biomaterials

Phototherapeutic biomaterials represent a rapidly advancing class of multifunctional systems that harness light, particularly in the near-infrared (NIR) window (≈700–1,000 nm), to achieve precise therapeutic outcomes (Overchuk et al., 2023; Jiang et al., 2026). These materials are engineered to respond to NIR irradiation through photothermal therapy (PTT), in which absorbed light is converted into localized heat, and photodynamic therapy (PDT), where light activation of photosensitizers induces ROS production (Jiang et al., 2026). Typical photothermal and photodynamic components include inorganic nanomaterials, carbon-based materials, metallic nanoparticles, conjugated polymers, and organic photosensitizers, which can be integrated into hydrogels, coatings, scaffolds, or injectable matrices depending on the target application. A key advantage of these platforms lies in their ability to deliver controlled, on-demand antimicrobial activity by disrupting bacterial membranes, denaturing proteins, and damaging nucleic acids. However, unlike conventional antibiotics, which distribute systemically and may contribute to resistance, phototherapeutic biomaterials remain inactive until triggered by an external stimulus, thereby confining their on-demand action to the target site (Jiang et al., 2026). Furthermore, these systems can be engineered for selective bacterial targeting, which enhances local phototoxicity while minimizing off-target effects on host tissue. This selectivity is commonly achieved through electrostatic interactions, enabling preferential binding of cationic photosensitizers to bacterial membranes, or though the incorporation of specific targeting moieties, such as antimicrobial peptides, carbohydrates, antibiotics, or even bacteriophages, that recognize bacterial surface receptors or cell wall components (Jiang et al., 2026). Beyond direct bacterial killing, the combination of PTT and PDT can also improve antibiofilm efficacy, as localized hyperthermia may disrupt the extracellular polymeric matrix and increase bacterial susceptibility to ROS-mediated damage. This synergistic effect is particularly relevant in implant-associated infections and chronic wounds, where biofilm formation strongly limits the efficacy of conventional antimicrobial treatments. Phototherapeutic biomaterials can also be leveraged to stimulate tissue repair and regeneration processes. PDT, for instance, can enhance acute inflammatory responses and stimulate wound closure (Ning et al., 2022; Selestin Raja et al., 2024). It also promotes angiogenesis and the deposition of ECM components, thereby supporting skin regeneration (Ning et al., 2022). In addition, both PDT and PTT can induce dose-dependent vascular effects, including increased blood flow and oxygenation, thereby improving hemoglobin saturation (Overchuk et al., 2023). Furthermore, both modalities are capable of activating immune responses, contributing to improved healing and host defense (Overchuk et al., 2023). In parallel, photobiomodulation itself may also provide therapeutic benefits. Low-intensity NIR light can stimulate mitochondrial activity, increase ATP production, and modulate cellular signaling pathways, leading to enhanced angiogenesis, fibroblast proliferation, and collagen synthesis, thereby further supporting tissue repair and regeneration (Selestin Raja et al., 2024). Despite these advantages, several challenges should be considered when designing phototherapeutic biomaterials. PDT efficacy may be reduced in hypoxic microenvironments because oxygen is required for ROS generation, whereas excessive photothermal heating can damage surrounding host tissues. Moreover, limited light penetration, non-uniform irradiation, and the need to optimize wavelength, power density, and exposure time remain critical parameters for clinical translation. Therefore, current strategies increasingly focus on integrating phototherapeutic agents into smart biomaterial platforms capable of improving local retention, enhancing bacterial targeting, modulating oxygen availability, and combining antimicrobial action with regenerative cues. In this context, multifunctional biomaterials integrating PTT and/or PDT have emerged as promising candidates for simultaneous infection control and tissue regeneration.

#### 3.3.1 Carbon-based phototherapeutic agents

Carbon-based photothermal agents, including graphene oxide, reduced graphene oxide, carbon nanotubes, and carbon nanodots are particularly suitable for infected wound repair (Gan et al., 2025) and bone regeneration applications (Cabral et al., 2024). Considerable effort has recently focused on tailoring their structure, surface chemistry, and composition, as well as on integrating them into hybrid nanocomposites and biomaterial matrices. For instance, the antimicrobial photothermal antibacterial activity of carbon dots (CDs) was combined with heat-enhanced antioxidant properties using a simple solvothermal method with inexpensive citric acid and ascorbic acid as precursors. Here, upon NIR irradiation, the CDs enabled efficient photothermal performance and showed broad-spectrum antibacterial activity against MRSA, *P. aeruginosa*, and *E. coli*. In parallel, residual ascorbic acid on the carbon dot surface endowed the nanoparticles with ROS-scavenging capacity. Notably, photothermal heating further enhanced their antioxidant performance, producing a synergistic effect through improved ROS elimination and stronger protection against oxidative injury (Wang H. et al., 2024). Photothermal conversion efficiency of carbon-based systems has also been improved through hybrid nanocomposite designs, such as Ag-decorated graphene oxide (Sun et al., 2024). Their incorporation into GelMA hydrogels or silk fibroin matrices not only significantly improved photothermal conversion efficiency and antibacterial activity against both *E. coli* and *S. aureus*, but also accelerated wound repair by stimulating collagen deposition, reducing inflammation and oxidative stress, and supporting wound closure through the combined action of carbon-based phototherapeutic systems and Ag release (Sun et al., 2024). Carbon-based materials can also impart electroconductivity to wound dressings, enabling the design of bioactive hydrogels that support endogenous electrical signal transmission during tissue regeneration. Xie et al. (2023) developed an electroactive NIR-responsive hydrogel by incorporating indocyanine green-loaded polydopamine-mediated graphene oxide and amorphous calcium phosphate into a poly (vinyl alcohol) (PVA) network. In this system, polydopamine promoted the reduction of graphene oxide, thereby conferring electroactivity to the hydrogel and providing abundant binding sites for indocyanine green loading. The resulting hydrogel exhibited NIR-triggered, on-demand antibacterial activity against *S. aureus* and *E. coli*. In addition, the NIR-induced thermal gel–sol transition of PVA promoted Ca^2+^ release and improved hydrogel adaptability to irregular wound shapes. The conductive and cell-affinitive properties of the graphene-based component further supported endogenous electrical signal transfer, thereby influencing cell behavior and promoting tissue regeneration both *in vitro* and *in vivo* (Xie et al., 2023).

#### 3.3.2 Metal-based phototherapeutic agents

Metal-based nanomaterials represent one of the most extensively explored groups of phototherapeutic biomaterials because they combine strong optical responses with intrinsic antimicrobial or regenerative ion activity. Noble-metal nanostructures, such as gold nanorods, efficiently convert NIR light into heat through localized surface plasmon resonance and can be functionalized with peptides, polymers, or targeting ligands to improve selectivity toward bacteria. A representative example is provided by antimicrobial peptide-functionalized gold nanorods, where conjugation with C-At5 enhanced binding to both Gram-positive and Gram-negative bacteria, reduced toxicity toward fibroblasts and red blood cells, and accelerated infected wound healing under laser irradiation (Xu X. et al., 2022). More recent gold nanorod hydrogel systems have combined PTT with additional chemical antibacterial mechanisms. For instance, a hyaluronic acid-dopamine and N-halamine-containing hydrogel loaded with gold nanorods displayed injectability, self-healing, tissue adhesion, hemocompatibility, and synergistic antibacterial activity through the combined effects of heat and active chlorine release, highlighting the increasing trend toward integrating plasmonic photothermal action with matrix-derived wound-healing functions (Wu et al., 2024). Silver and copper-based photothermal platforms further illustrate the multifunctionality of metal-containing systems. Silver nanoparticles provide intrinsic broad-spectrum antibacterial activity and can cooperate with photothermal or photodynamic components to enhance bacterial killing at lower doses (Cao et al., 2024). Copper-containing photothermal agents, including copper sulfide and copper oxide nanostructures, are especially attractive because they combine NIR responsiveness with the biological activity of Cu^2+^ ions, which can stimulate angiogenesis and contribute to tissue remodeling. In PAG-CuS hydrogels, CuS nanoparticles enabled photothermal sterilization, while staged release of lipoic acid sodium salt regulated ROS and MMP-9 levels and Cu^2+^ release promoted CD31 expression, microvessel formation, epithelialization, and diabetic wound repair (He et al., 2024). Similarly, biomimetic hydrogels containing CuS nanoparticles and angiogenic regulators have been used to combine photothermal antibacterial activity with extracellular matrix-mimetic support and HIF-1α/VEGF-associated vascular stimulation, underscoring the ability of copper-based photothermal platforms to address both bacterial burden and ischemic, poorly vascularized wound environments (Shen et al., 2024).

MOFs add a further level of control, as they can act as photothermal agents, photodynamic platforms, drug reservoirs, and ion-releasing components, while their degradation can be tuned by pH or other infection-associated cues. Huang et al. developed a quaternized chitosan MOF-vancomycin hydrogel that captured bacteria and, under NIR irradiation, maintained mild hyperthermia around 43 °C. This temperature was sufficient to synergize with Zn^2+^ and vancomycin for bacterial killing while avoiding excessive thermal injury to host tissue. Beyond antimicrobial activity, the hydrogel modulated the M1/M2 macrophage balance and promoted nerve and vascular regeneration in infected wounds (Huang K. et al., 2022). MOF-based hydrogels have also been developed for PDT-dominated treatment; photodynamic alginate Zn-MOF thermosensitive hydrogels, for example, provided sustained photosensitizer delivery and light-triggered antibacterial activity while accelerating closure of infected wounds (Zhang W. et al., 2023). The same design logic has been translated into mineralized and dental regenerative contexts. Qiu et al. (2023) incorporated SrCuSi_4_O_10_ into GelMA hydrogels to create a photothermal pulp-capping platform. Under NIR irradiation, the composite efficiently eliminated oral pathogens such as *Streptococcus mutans* and *Lactobacillus casei* and inhibited biofilm formation, while released ions promoted odontogenic differentiation of dental pulp stem cells and angiogenic behavior of endothelial cells. This example is particularly relevant because it demonstrates that phototherapeutic biomaterials can be designed not only for skin wound healing but also for infection-resistant regeneration of specialized tissues, where antibacterial activity must be balanced with lineage-specific differentiation and vascular support (Qiu et al., 2023).

#### 3.3.3 Semiconductor and two-dimensional phototherapeutic materials

Semiconductor and two-dimensional nanomaterials, including MoS_2_, WS_2_, black phosphorus (BP), and MXenes, have attracted increasing attention as multifunctional antibacterial and regenerative platforms. Their high NIR absorbance, tunable band structures, large surface area, and capacity for surface functionalization allow them to generate heat, ROS, or both, depending on composition and irradiation conditions. Transition-metal dichalcogenides such as MoS_2_ can be stabilized in chitosan- or hyaluronic acid-based hydrogels to provide injectable, adhesive, and self-healing matrices with photothermal antibacterial activity. In recent MoS_2_-functionalized hydrogels, NIR-triggered heating was combined with antioxidant and hemostatic functions, allowing bacterial killing while reducing oxidative stress and supporting cell compatibility (Lu et al., 2024; Wang P. et al., 2024). Such systems are particularly valuable because infected wounds often require photothermal doses that would be damaging if used alone; by adding antioxidant or catalytic functions, the biomaterial can reduce inflammatory injury while maintaining antibacterial efficacy. MXenes have emerged as another important class of photothermal two-dimensional materials. Their strong NIR absorption, electrical conductivity, hydrophilicity, and ion-intercalation capacity make them suitable for wound dressings, bioelectronic interfaces, and tissue-regenerative scaffolds. Cu(II)-coordinated MXene hydrogels, for instance, have been shown to act simultaneously as photothermal antibacterial barriers, ROS scavengers, and angiogenesis-promoting platforms. *In vivo*, these systems improved inflammatory resolution, collagen deposition, vessel formation, and wound closure in infected wounds, illustrating how MXene-based materials can bridge antimicrobial action, redox regulation, and vascular repair (Liu et al., 2023). More recently, MXene nanocomposite hydrogels have also been investigated for diabetic wounds, where photothermal bacterial killing is complemented by enhanced cell proliferation and migration, suggesting that the conductive and mechanically reinforcing roles of MXenes can be exploited together with their optical properties to support regeneration in metabolically impaired tissues (Ou et al., 2025).

Among emerging two-dimensional semiconductors, BP deserves particular attention because it combines strong NIR absorption, photothermal and photodynamic activity, biodegradability, and biologically favorable degradation products. BP nanosheets and quantum dots gradually oxidize into phosphate-containing species under physiological conditions, which can support mineralization and bone repair while reducing long-term persistence concerns (Bigham et al., 2026). This property makes BP-based biomaterials especially attractive for infected bone defects, where antimicrobial therapy must be coordinated with osteogenesis (Miao et al., 2019; Yuan et al., 2022; Jing et al., 2023; Li et al., 2024). In parallel, BP hybrid hydrogels and scaffolds have been reported to support photothermal antibacterial activity, drug release, collagen deposition, neovascularization, and inflammatory regulation in difficult-to-treat diabetic infected wounds (Xu S. et al., 2021; Huang S. et al., 2022; Yu et al., 2026). Beyond NIR-responsive PTT capability, he incorporation of semiconductor nanosheets has also recently been to impart electrical conductivity, thereby broadening their role in tissue regeneration (Jing et al., 2023; Wang R. et al., 2025). Indeed, a BP-based conductive system has been recently explored to engage the neural component of bone repair. In this study, the authors developed a photosensitive and conductive GelMA-based hydrogel containing BP and Mg^2+^ for infected skull defect repair (Figure 3A). Upon NIR irradiation, BP-mediated photothermal and photodynamic effects were observed (Figure 3B) and conferred potent antibacterial activity against *Escherichia coli* and *S. aureus* (Figure 3C), improved the inflammatory microenvironment, and reduced bacteria-induced bone tissue damage. Meanwhile, the conductive nanosheets and released bioactive ions acted synergistically to enhance Schwann cell migration and secretory activity, stimulate neurite outgrowth, and support innervated bone regeneration (Figure 3D), while simultaneously enhancing bone regeneration (Figure 3E) (Jing et al., 2023).

**FIGURE 3:**
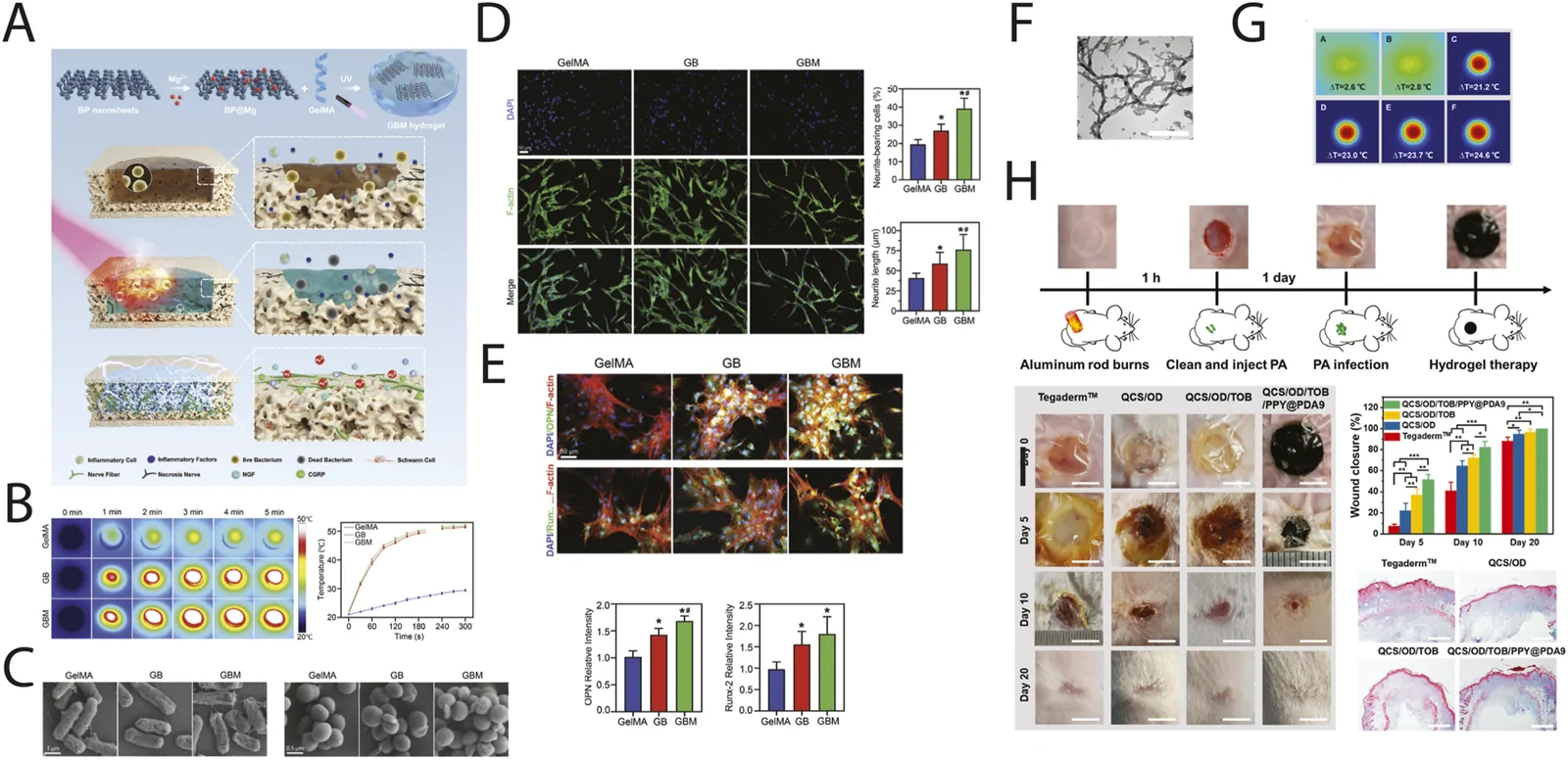
Photothermal platforms for antibacterial therapy and tissue regeneration. **(A)** Schematic illustration of GelMA, GelMA and BP (GB), and GelMA, BP and Mg (GBM) hydrogel fabrication and their application in the regeneration of infected bone defects. Under NIR irradiation, the GBM hydrogel exhibits strong antibacterial activity, reducing bacteria-induced damage to bone tissue. Magnesium-modified black phosphorus nanosheets promoted skeletal-associated nerve fiber repair and accelerated bone regeneration. **(B)** On the left, infrared thermographic images of GelMA, GB, and GBM hydrogels under NIR irradiation. On the right, temperature changes of the hydrogels during 808 nm NIR laser irradiation confirming PTT effect. **(C)** SEM images of *E. coli* and *S. aureus* after NIR irradiation following treatment with GelMA, GB, or GBM hydrogels. **(D)** On the left, immunofluorescence images of PC12 cells cultured on different hydrogels; cells with protrusion lengths greater than 10 μm were defined as neurite-bearing cells. On the right, quantitative analysis of the percentage of neurite-bearing cells and neurite length. **(E)** Immunofluorescence staining images of Osteopontin (OPN) and Runx-2 in MSCs cultured on GelMA, GB, and GBM hydrogels. The corresponding quantitative analysis of OPN and Runx-2 fluorescence intensity is shown below. Adapted from (Jing et al., 2023) with permission from Wiley. **(F)** TEM image of PDA-coated PPy nanowires. Scale bar: 500 nm. **(G)** Infrared thermal images of QCS/OD/TOB/PPY/PDA hydrogels under 808 nm NIR irradiation. **(H)** Schematic illustration of the construction of the *P. aeruginosa*-infected burn wound model. **(I)** On the left, representative wound photographs after treatment with QCS/OD/TOB/PPY/PDA hydrogel. Scale bar: 5 mm. Right: Quantitative evaluation of wound closure and Masson’s trichrome staining images. Scale bar: 800 μm. Adapted from (Huang Y. et al., 2022) with permission from American Chemical Society.

#### 3.3.4 Polymer-based phototherapeutic systems

Polymer-based phototherapeutic systems are highly relevant because they combine efficient light-to-heat conversion with processability, biocompatibility, and tissue-mimetic mechanics. Conductive and catechol-containing polymers such as polydopamine (PDA), polypyrrole (PPy), and polyaniline can be incorporated into hydrogels, coatings, electrospun membranes, or porous scaffolds to generate light-responsive antibacterial interfaces. PDA is particularly versatile: beyond its broad NIR absorption and photothermal conversion, its catechol and quinone groups provide wet adhesion, metal chelation, radical scavenging, and easy secondary functionalization (Huang Y. et al., 2022; Ren et al., 2022; Zhao P. et al., 2023). These properties are useful in infected wounds because stable adhesion ensures close contact between the dressing and irregular wound surfaces, while antioxidant activity helps counteract infection-associated oxidative stress (Huang K. et al., 2022; Chen et al., 2023; Zhao P. et al., 2023). PDA has also been combined with other functional polymers to generate more complex, multifunctional antimicrobial and wound-healing systems. For example, in a quaternized chitosan/oxidized dextran/tobramycin/PPy/PDA hydrogel, PDA was used as a multifunctional coating on conductive polypyrrole nanowires (Figure 3F), facilitating their integration into a biologically active polymeric matrix (Huang Y. et al., 2022). In this design, the different polymeric components played complementary roles: quaternized chitosan contributed antibacterial activity and biocompatibility, oxidized dextran enabled dynamic crosslinking and self-healing behavior, PPy provided electrical conductivity and strong NIR-responsive photothermal activity, while the PDA shell improved the biological interface of the nanowires and introduced catechol/quinone-mediated functions. Overall, PDA contributed antioxidant activity, enhanced the compatibility of PPy within the hydrogel network, and supported NIR-assisted photothermal antibacterial performance. Under NIR irradiation, the PPy/PDA nanowires generated localized heat (Figure 3G), which enhanced bacterial killing and acted synergistically with bacteria-responsive tobramycin release, thereby accelerating closure of *P. aeruginosa*-infected burn wounds (Figures 3H,I) (Huang Y. et al., 2022).

Polymer-based photothermal systems have also been combined with photodynamic or gas-releasing strategies to enhance biofilm disruption and immune regulation. Chen et al. (2023) developed CaSiO_3_–ClO_2_ nanoparticles functionalized with polydopamine and indocyanine green. In acidic infected environments, ClO_2_ was released and disrupted the biofilm structure, enhancing nanoparticle penetration and antibacterial activity against *E. coli* and *S. aureus*. Upon NIR irradiation, polydopamine-mediated photothermal heating and indocyanine green-mediated ROS generation were simultaneously activated, leading to enhanced antibacterial efficacy. In addition to direct bacterial killing, this combined PDT/PTT strategy stimulated immune activation and promoted macrophage phenotype switching, thereby linking antimicrobial therapy with inflammation regulation and tissue regeneration (Chen et al., 2023).

### 3.4 Nanoenzyme-based biomaterials

Nanozymes are nanomaterials that possess inherent enzyme-like catalytic activity, acting as artificial enzymes. Beyond merely mimicking natural enzymes, they integrate nanoscale physical characteristics with advantages such as high stability, cost-effectiveness, and adjustable activity, enabling their application in biomedical and environmental fields (Jiang et al., 2019). More recently, nanozyme-based biomaterials have evolved from simple catalytic agents into multifunctional, microenvironment-responsive platforms designed to first suppress infection and subsequently promote tissue regeneration by dynamically modulating redox balance, oxygenation, inflammation, and ECM remodeling (Jiang et al., 2019).

Endogenous cues from infected tissues can be used by nanozymes to implement this staged catalytic program. These include pH gradients, glucose levels, ROS concentration, and infection-associated wound exudates, which initially activate ROS-generating nanozyme functions for effective bacterial eradication. Subsequently, either through intrinsic responsiveness or integrated multi-component design, the system shifts toward ROS-scavenging and oxygen-generating activities, thereby facilitating macrophage repolarization, angiogenesis, cell proliferation, and ECM remodeling. Accordingly, the central advance in the field lies in the development of programmable or self-adaptive nanozymes capable of dynamically transitioning between these functional states, enabling this “kill first, then heal” paradigm. The catalytic activities most relevant for coupling infection control with tissue regeneration include peroxidase (POD)-like, oxidase (OXD)-like, catalase (CAT)-like, and superoxide dismutase (SOD)-like functions. POD-like and OXD-like nanozymes are primarily exploited for antibacterial activity through ROS generation. POD-like nanozymes catalyze the conversion of hydrogen peroxide into highly cytotoxic hydroxyl radicals under acidic or Fenton-like conditions, whereas OXD-like systems directly generate ROS from molecular oxygen without requiring exogenous H_2_O_2_. In contrast, SOD-like and CAT-like activities are particularly valuable for restoring redox balance and improving the regenerative microenvironment. SOD-like nanozymes catalyze the dismutation of superoxide radicals into O_2_ and H_2_O_2_, while CAT-like nanozymes further decompose H_2_O_2_ into water and oxygen, thereby reducing oxidative damage and alleviating tissue hypoxia (Jiang et al., 2019; Zhang et al., 2019; Liu X. et al., 2025; Xing et al., 2025), the latter two activities play a disproportionately important role in chronically infected pathological conditions, including chronic wounds, diabetic ulcers, periodontitis, and ischemic flaps, where successful regeneration depends not on complete ROS elimination, but on restoring ROS levels to a tightly regulated, signaling-compatible window (Liu X. et al., 2025; Xing et al., 2025). A key design principle underpinning these systems is temporal and functional adaptability. During the early, infection-dominated phase, typically characterized by acidic pH and high bacterial load, POD-like and oxidase-like activities are preferentially activated to generate bactericidal ROS. As the microenvironment transitions toward a healing phase, CAT-like and SOD-like activities become dominant, decomposing excess hydrogen peroxide, mitigating oxidative damage, improving local oxygen supply, and restoring a pro-regenerative milieu (Jiang et al., 2019; Zhang et al., 2019; Liu X. et al., 2025; Xing et al., 2025). From a materials perspective, recent classifications categorize nanozymes into several major groups, including metal- and metal oxide-based nanozymes, carbon-based, MOF-based nanozymes, and single-atom nanozymes (Jiang et al., 2019; Jiang et al., 2019; Jiang et al., 2019). In parallel, increasingly sophisticated hybrid systems have also been developed by integrating nanozymes into hydrogels, scaffolds, membranes, microneedles, and surface coatings to improve catalytic efficiency, therapeutic retention, and microenvironment responsiveness (Jiang et al., 2019).

#### 3.4.1 Metal- and metal-oxide nanozymes

Metal-based nanozymes represent a prominent class of catalytic nanomaterials owing to their rich redox chemistry and tunable catalytic behavior. A central feature of many metal-based nanozymes is their ability to mediate Fenton and Fenton-like reactions, which underpin their POD-like activity (Tian et al., 2023; Xu et al., 2023; Cui et al., 2024; Wang X. et al., 2026). Cui and co-workers developed a Cu nanodots/cationic guar hydrogel that leverages the POD-like activity of Cu nanodots to combat *E. coli* and *S. aureus*, enabling biofilm disruption and potent antibacterial performance. In parallel, the controlled release of Cu ions reduced inflammatory marker expression and promoted angiogenesis (Cui et al., 2024). To further improve POD-like bactericidal activity, Wang et al., developed a heme–Cu coordinated nanozyme via the co-assembly of amino acids with Cu ions to replicate the catalytic microenvironment of natural peroxidases. This biomimetic architecture significantly enhanced POD-like bactericidal activity against *E. coli* and *S. aureus* under acidic, infection-associated conditions, while allowing a transition to SOD- and CAT-like cascade activity at neutral pH, thereby reducing oxidative stress and supporting tissue recovery and angiogenesis. Incorporation of the nanozyme into GelMA hydrogels further improved cytocompatibility, enabled sustained release, and preserved wound moisture (Wang X. et al., 2026). Recent efforts have focused on refining spatiotemporal control over nanozyme delivery. In this regard, triglycerol monostearate-based systems offer a promising strategy for responsive release. Specifically, Cu–tannic acid nanozymes have been incorporated into a triglycerol monostearate/2,6-di-tert-butyl-4-methylphenol hydrogel. Due to the presence of ester linkages, this hydrogel exhibits enzyme-responsive degradability, undergoing cleavage in esterase- and matrix metalloproteinase-9-rich inflammatory environments. This property enables precise, on-demand nanozyme release at diseased sites. Once released, the Cu–tannic acid nanozyme exerts antibacterial and anti-biofilm effects against *E. coli* and *S. aureus*, while also regulating macrophage polarization, suppressing inflammation, and promoting osteogenesis in periodontitis models (Xu et al., 2023). To further improve localized delivery, microneedle-based platforms for nanozyme delivery have also been explored. For example, a Zn–gallic acid nanozyme was integrated into a gelatin-based microneedle patch to enable transdermal and sustained delivery at infected wound sites. The Zn-based nanozyme showed effective antibacterial activity against *E. coli* and *S. aureus*, along with strong antioxidant capacity and minimal cytotoxicity. The microneedle system facilitated penetration through the epidermis, efficient biofilm disruption, and localized therapeutic action, while simultaneously reducing oxidative stress, suppressing inflammation, and promoting angiogenesis (Dong et al., 2024). Although these strategies have demonstrated remarkable efficacy against common wound pathogens, the treatment of Gram-negative bacterial infections remains particularly challenging because of the protective outer membrane and highly adaptive defense mechanisms characteristic of these microorganisms. Consequently, increasing attention has been directed toward engineering metal-based nanozyme systems capable of selectively targeting Gram-negative pathogens and overcoming their intrinsic resistance mechanisms. In this context, a multifunctional Cu_x_Te nanozyme was incorporated in a cationic guar gum hydrogel to develop a selective antibacterial platform against Gram-negative pathogens. In this system, Cu_x_Te nanozymes were incorporated into a biocompatible and adhesive cationic guar gum matrix, combining the catalytic properties of the nanozymes with the favorable physicochemical characteristics of the hydrogel. The hydrogel exhibited synergistic OXD- and glutathione peroxidase-like activities, promoting ROS generation while simultaneously depleting intracellular glutathione, thereby inducing severe oxidative stress in Gram-negative bacterial cells. Importantly, transcriptomic analysis revealed that the platform selectively targeted *P. aeruginosa* by downregulating genes involved in lipopolysaccharide biosynthesis and flagellar assembly, impairing both membrane integrity and bacterial motility (Figure 4A). The disruption of these critical protective and virulence-associated structures further amplified ROS-mediated antibacterial activity, resulting in efficient eradication of Gram-negative pathogens, including *E*. *coli*, *P*. *aeruginosa*, and *Klebsiella pneumoniae*, as well as effective biofilm inhibition and disruption (Figures 4B,C). Furthermore, in a *P*. *aeruginosa*-infected burn wound model, the hydrogel significantly accelerated wound healing by eliminating bacterial infection, promoting angiogenesis, and modulating inflammatory responses, while maintaining excellent biosafety (Niu et al., 2026; Niu et al., 2026; Niu et al., 2026). Metal-oxide nanozymes derive their catalytic versatility from variable valence states and oxygen vacancies, which regulate substrate adsorption, electron transfer, and redox cycling (Yousaf et al., 2025; Hu S. et al., 2023; Xue et al., 2024). This intrinsic redox tunability has positioned them as promising platforms for the simultaneous control of infection and promotion of tissue regeneration. For instance, the OXD-like activity of MnO_2_ nanozyme has been successfully used to eliminate *S. aureus* and *E. coli* by disrupting biofilm, leading to superior antibacterial efficacy *in vivo* compared to vancomycin. At the same time, MnO_2_ nanozyme was shown to accelerate wound healing in murine models, highlighting its strong potential for applications in skin and soft tissue infection management (Liu et al., 2021). To improve the therapeutic performance of metal-oxide nanozymes, considerable efforts have been directed toward overcoming nanoparticle aggregation and enhancing colloidal stability, as these factors critically influence catalytic activity and biological interactions. In this context, CeO_2_ nanoparticles coated with Pluronic F127 were developed to improve nanozyme dispersion, stability, and therapeutic efficacy. Compared to uncoated CeO_2_ nanoparticles, the coated nanozymes exhibited reduced aggregation, enhanced stability, and improved catalytic activity. Owing to their larger effective surface area and increased Ce^3+^/Ce^4+^ ratio, the nanoparticles promoted enhanced oxygenation and significantly increased angiogenesis, while demonstrating efficient antimicrobial activity against *P. aeruginosa*, resulting in accelerated healing of chronically infected wounds (Padaga et al., 2025). Metal-oxide nanozymes have also been integrated into hydrogel matrices to combine enzyme-mimetic catalytic activity, controlled therapeutic metal ion release, and prolonged wound-site retention within a single platform (Zhou et al., 2025; Wang J. et al., 2026). For instance, CuO_2_ nanozymes were incorporated into a thiol-modified hyaluronic acid matrix to generate a multifunctional disulfide-crosslinked hydrogel. The resulting hydrogel exhibited strong tissue adhesiveness, high water-retention capacity, and self-healing properties, enabling stable localization of the nanozymes at the wound site. Under the mildly acidic conditions characteristic of infected wounds, the embedded CuO_2_ nanozymes displayed pronounced POD-like catalytic activity together with Cu^2+^ ion release. Here, the synergistic effects of ROS generation and Cu-induced cuproptosis-like mechanisms resulted in highly efficient *S. aureus* eradication and enhanced wound closure, reduced inflammatory responses, increased collagen deposition, and promoted epidermal regeneration in an *in vitro* infected wound model (Wang J. et al., 2026). Similarly, Cu–Fe_3_O_4_ nanocomposite-loaded hyaluronic acid methacrylate hydrogels combined OXD-like and glutathione-like catalytic activity with Cu-mediated antibacterial effects, enabling efficient eradication of *E. coli* and S*. aureus* while promoting wound healing within 14 days after patch application (Zhou et al., 2025). In other approaches, ROS-scavenging CeO_2_ nanozyme activity has been combined with vancomycin-mediated antibacterial therapy against *P. aeruginosa* and *S. aureus* through multifunctional hybrid hydrogels (Zhao S. et al., 2024). More sophisticated “all-in-one” nanozyme systems have additionally been developed to couple antibacterial therapy with subsequent ROS scavenging and tissue regeneration. Specifically, the photosensitizer chlorin e6 was conjugated with a CeO_2_ nanozyme. Upon red-light irradiation, Ce6 generates a rapid burst of ROS for efficient bacterial eradication. Subsequently, the nano-octahedral CeO_2_ component, characterized by a high surface concentration of Ce^3+^, acts as a potent ROS scavenger, restoring redox balance. This dynamic regulation between ROS production and clearance not only ensures antibacterial efficacy but also modulates macrophage polarization, reducing inflammation and promoting tissue regeneration (Sun et al., 2021). These concepts have been further extended into multifunctional hybrid platforms. For instance, a mussel-inspired adhesive hydrogel reinforced with MnO_2_ nanoparticles was developed by integrating polyvinyl alcohol (PVA) with 3,4-dihydroxy-L-phenylalanine (DOPA). The incorporation of MnO_2_ imparts SOD- and CAT-like activities, alleviating hypoxia and modulating the inflammatory microenvironment. In synergy with DOPA, MnO_2_ also enhances photothermal antibacterial and antibiofilm performance against *E. coli*, *S. aureus*, and *Porphyromonas gingivalis*. In a periodontitis model, this system not only suppresses infection and oxidative stress but also promotes bone regeneration and tissue regeneration (Hu S. et al., 2023).

**FIGURE 4:**
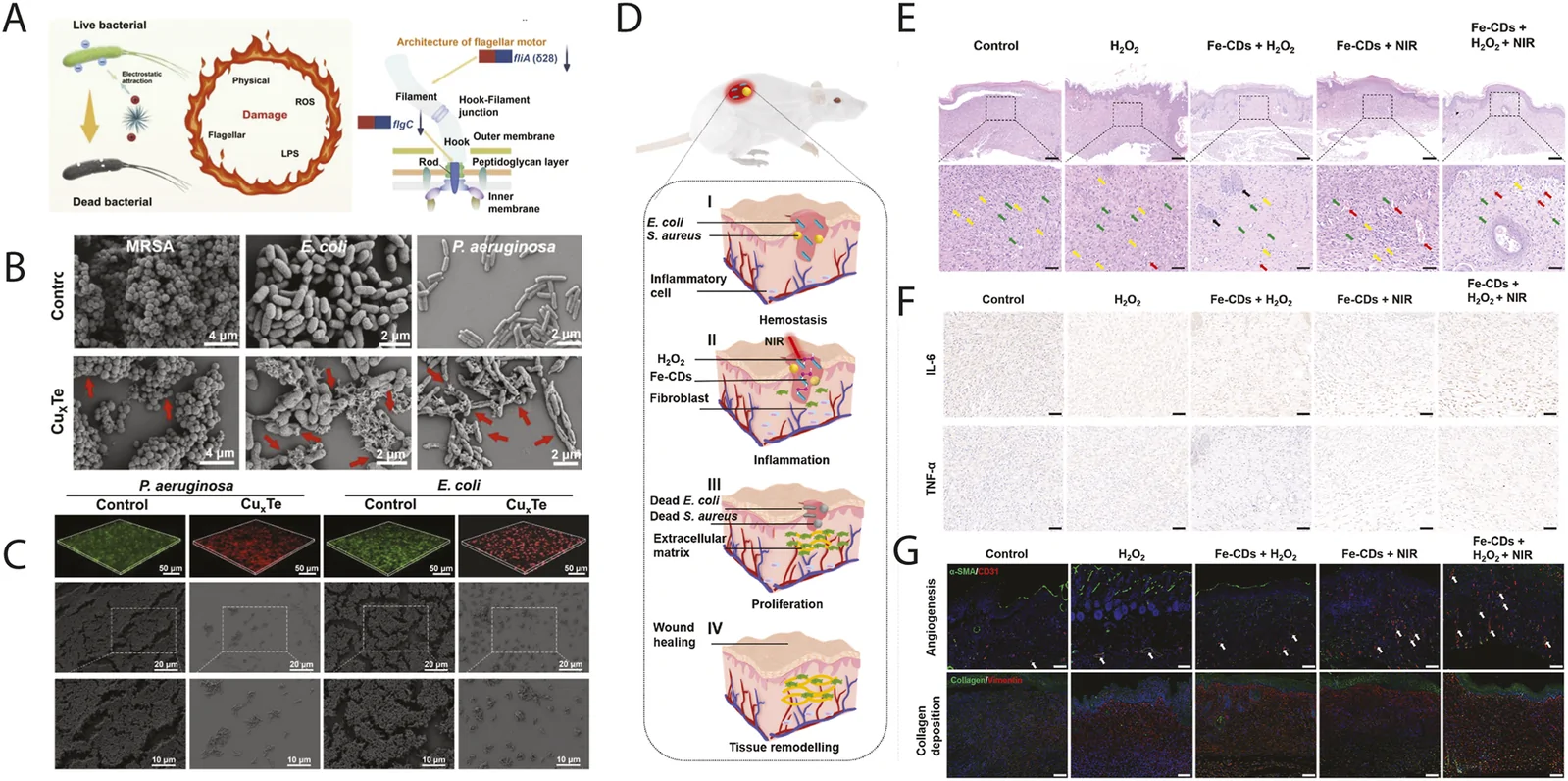
Nanozyme-Based Antibacterial Strategies and Their Therapeutic Applications. **(A)** Schematic representation of the antibacterial mechanism of CuxTe nanozymes and their effect on *P. aeruginosa* flagellar assembly. **(B)** SEM images of MRSA, *E. coli*, and *P. aeruginosa* treated with CuxTe nanozymes at 20 μg/mL compared with the control group. **(C)** Representative 3D reconstruction images of *P. aeruginosa* and *E. coli* biofilms in the CuxTe-treated group (20 μg/mL) and control group. Scale bar: 50 μm. Below are representative SEM images of *P. aeruginosa* and *E. coli* biofilms under the same conditions. Adapted from (Niu et al., 2026) with permission from JPL. **(D)** Schematic illustration of the Fe-CDs-based therapeutic platform for wound-healing applications. **(E)** Representative H&E-stained images of dermal wounds. Yellow arrows indicate inflammatory cells, including lymphocytes, neutrophils, and macrophages; black arrows indicate hair follicles; green arrows indicate fibroblasts; and red arrows indicate newly formed blood vessels. Scale bar: 200 μm; magnified images: 50 μm *p < 0.05, **p < 0.01. **(F)** Immunohistochemical staining images of IL-6 and TNF-α in granulation tissue on day 10. Scale bar: 50 μm. **(G)** Double immunofluorescence staining images showing α-SMA (green) and CD31 (red), as well as collagen (green) and vimentin (red). Scale bar: 100 μm. Adapted from (Liu et al., 2022) with permission from Elsevier.

#### 3.4.2 Carbon-based nanozymes

Carbon-based nanozymes are synthetic nanomaterials built upon carbon frameworks that exhibit tunable catalytic activity, and exceptional physicochemical stability. The diverse sp^2^/sp^3^ hybridization configurations of carbon confer versatile structural characteristics enabling precise modulation of enzyme-mimetic behavior. Carbon-based nanozymes exert antimicrobial effects primarily through POD-like ROS generation, synergistic photothermal bactericidal activity, or enhanced biofilm penetration. These systems are particularly attractive for biomedical and tissue regeneration applications owing to their good biocompatibility, potential biodegradability, and, in metal-free systems, minimal risk of metal ion leakage (Zhuang et al., 2024; Liu X. et al., 2025; Tripathi et al., 2025). However, the intrinsic catalytic activity of pure carbon frameworks is often limited, necessitating strategies such as defect engineering, heteroatom doping, or hybridization with catalytic metal species to achieve sufficient performance. Consequently, increasingly sophisticated hybrid nanozyme systems have been developed in which carbon phases function not only as structural carriers, but also as active regulators of electron transfer and redox kinetics. In addition to enhancing antibacterial activity, heteroatom doping has also been employed to impart antioxidant and pro-angiogenic functions to carbon-based nanozymes. In one representative study, multifunctional selenium–sulfur co-doped carbon dot nanozymes were synthesized using selenocysteine and cysteine precursors to simultaneously target several pathological hallmarks of infected wounds. These nanozymes exhibited potent antibacterial activity by disrupting bacterial membrane integrity, leading to efficient pathogen elimination at infected wound sites. Simultaneously, the dynamic Se–S bonds selectively activated the thioredoxin reductase pathway, thereby effectively scavenging excess ROS and alleviating inflammatory responses. Moreover, the doped carbon dots promoted angiogenesis through upregulation of pro-angiogenic signaling pathways, resulting in enhanced neovascularization and accelerated tissue regeneration (Huang et al., 2026). Metal doping has also proven highly effective in improving carbon-based nanozymes with synergistic PTT and chemodynamic therapy capabilities, thereby further enhancing their antibacterial and wound-healing performance. For example, Fe-doped carbon dots synthesized via a one-pot pyrolysis strategy demonstrated significantly enhanced photoactivated POD-like catalytic activity together with excellent photothermal conversion capability compared with pristine carbon dots. Under NIR irradiation, these nanozymes efficiently eradicated *S. aureus* and *E. coli* while minimizing damage to surrounding tissues. Importantly, localized hyperthermia further amplified Fe-doped carbon dot-mediated catalytic ROS generation, thereby enhancing antibacterial efficacy and accelerating tissue regeneration through increased fibroblast proliferation, neovascularization, and collagen deposition (Figures 4D–G) (Liu et al., 2022). Building upon these advances, cascade nanozyme systems have also been developed to further improve therapeutic outcomes. Shen et al. (2023) developed a glucose-responsive photothermal nanozyme system consisting of glucose oxidase and Fe-doped carbon dots integrated within mesoporous polydopamine. In this cascade platform, glucose oxidase first catalyzes glucose oxidation to generate H_2_O_2_, which then serves as a substrate for the POD-like activity of Fe-doped carbon dots. Upon NIR irradiation, the nanozyme generated mild localized hyperthermia, enhancing bacterial membrane and biofilm permeability without harming surrounding healthy tissue. The combination of PTT and chemodynamic therapy (CDT) produced a synergistic antibacterial effect, enabling efficient healing of MRSA-infected diabetic wounds *in vivo* within 12 days (Shen et al., 2023). To further enhance therapeutic efficacy and improve local retention at wound sites, carbon-based nanozymes have also been integrated into hydrogel matrices, thereby combining the catalytic and photothermal properties of nanozymes with the favorable physicochemical characteristics of hydrogels. These hybrid systems provide moist wound environments, improved biocompatibility, controlled therapeutic delivery, and enhanced interaction with damaged tissues, making them highly attractive for infected wound management and tissue regeneration (Zhuang et al., 2024; Liu X. et al., 2025; Tripathi et al., 2025). Accordingly, a growing number of nanozyme-loaded hydrogel platforms have been engineered to exploit the synergistic integration of catalytic antibacterial activity, PTT, and tissue-regenerative functions within a single therapeutic system. In this context, Wu and co-workers constructed a graphdiyne–Fe nanozyme hydrogel by integrating a dopamine-modified hyaluronic acid matrix with a graphdiyne-Fe nanozyme. Under NIR irradiation, this multifunctional platform exhibited synergistic chemo-photothermal antibacterial activity against *E. coli*, *S. aureus*, and *P. gingivalis*. Beyond its potent antibacterial capability, the hydrogel also reduced inflammation and promoted tissue regeneration demonstrating therapeutic efficacy in both infected wound and rat periodontitis models (Wu et al., 2025). Similarly, electrochemically exfoliated graphene oxide nanosheets were incorporated into an alginate-based hydrogel, enabling a synergistic dual-mode antibacterial effect through ROS-induced oxidative stress and PTT upon NIR irradiation. Notably, these carbon-based nanozymes exhibited triple enzyme-mimetic activity: while the OXD- and POD-like activities promoted ROS generation and antibacterial action, the additional CAT-like activity of the carbon-based nanozyme system enabled ROS modulation and inflammation control, thereby creating a more favorable microenvironment for tissue regeneration. Consequently, this tri-enzyme-mimicking hydrogel effectively eliminated bacterial infection and accelerated wound healing in a mouse wound infection model (Qiao et al., 2025).

Metal oxide-based nanozymes have also demonstrated significant potential in the control of biofilms as they offer distinct advantages: they can generate reactive intermediates at the biofilm interface, deplete intracellular antioxidants such as glutathione, disrupt quorum-sensing pathways, and enhance penetration into biofilm matrices when integrated into deformable delivery platforms such as hydrogels or microneedles. A notable example is the photothermal nanozyme microneedle patch developed by Zhu et al. (2022), constructed by integrating Fe_3_O_4_ nanoparticles onto graphene oxide nanosheets and encapsulating them within methacrylated hyaluronic acid microneedles. Within the biofilm microenvironment, the nanozyme catalyzes the generation of hydroxyl radicals, which disrupt bacterial heat-shock proteins and sensitize the biofilm to thermal stress. Upon mild photothermal stimulation, enhanced iron uptake leads to intracellular iron overload, triggering ferroptosis-like bacterial cell death (Zhu et al., 2022). Antibiofilm efficacy of the photothermal nanozyme microneedle patch was also evaluated *in vivo* using both implant-associated and diabetic wound biofilm infection models, revealing complete biofilm removal, increased expression of pro-healing markers, angiogenesis and collagen deposition, indicative of effective tissue regeneration (Zhu et al., 2022).

#### 3.4.3 MOF-based nanozymes

MOFs occupy a particularly advantageous position among nanozyme platforms, as their highly porous architectures enable efficient loading of enzymes, therapeutic agents, or growth-factor analogues, while their tunable coordination environments facilitate the construction of biomimetic catalytic sites and stimuli-responsive release profiles. This structural versatility has enabled the development of integrated systems that combine catalytic activity with therapeutic delivery and microenvironment modulation. For instance, in a recent work, Wei et al. (2024) developed a dual-metal MOF nanozyme functionalized with the antibacterial agent chlorogenic acid and incorporated into a genipin-crosslinked chitosan hydrogel. In this design, chlorogenic acid is not merely physically loaded but participates in coordination with the metal nodes, contributing to the structural and catalytic functionality of the MOF nanozyme. The incorporated dual-metal MOF nanozymes exhibited CAT-like activity, enabling the conversion of elevated endogenous hydrogen peroxide into dissolved oxygen, thereby simultaneously alleviating oxidative stress and hypoxia in ulcerated wounds. In parallel, the antimicrobial action of chlorogenic acid suppressed infection by *E. coli* and *S. aureus* (Wei et al., 2024). This example highlights how MOF platforms can integrate catalytic, antimicrobial, and microenvironment-regulating functions within a single system. In a different approach, a nanocatalytic platform based on ZIF-8–Cu/C-dots was developed. In this system, ultrasmall carbon dots supporting Cu single atoms with strong antioxidant activity were used as ligands within a Zn^2+^-coordinated MOF framework. The Cu/C-dots exhibited enzyme-like antioxidant activity, OXD-like activity, reducing ROS-induced damage and downregulating pro-inflammatory cytokines, while Zn^2+^ provided strong antibacterial effects. *In vivo*, this system effectively alleviated inflammation, promoted angiogenesis, enhanced collagen deposition, and supported tissue remodeling in diabetic wounds (Dai et al., 2024).

#### 3.4.4 Single-atom nanozymes

Single-atom nanozymes offer not only maximized atom utilization but also precise control over catalytic pathways and active-site coordination, thereby enhancing both activity and selectivity. In a recent study, a Fe single-atom nanozyme was synthesized via a simple one-pot hydrothermal method and showed strong POD-like bactericidal activity against *E. coli* and *S. aureus*. In addition, the coexistence of Fe^2+^ and Fe^3+^ within the nanozyme also imparted anti-inflammatory properties, downregulating pro-inflammatory cytokines such as TNF-α and IL-1β while upregulating growth factors including VEGF and epidermal growth factor. This dual functionality not only controls infection and inflammation but also promoted fibroblast proliferation and accelerated wound closure (Chen S. et al., 2024).

Single-atom nanozymes can also be engineered to dynamically switch between distinct enzyme-mimetic activities in response to changes within the wound microenvironment, thereby enabling adaptive and stage-specific therapy. In this context, a pH/ROS dual-responsive Ce single-atom nanozyme spray was developed to intelligently adapt to the evolving microenvironment of infected wounds. During the early stages of infection, the nanozyme responded to the acidic wound environment by exhibiting cascade glucose oxidase (GOD)- and POD-like activities, thereby generating high levels of ROS and exerting potent antibacterial effects against *S. aureus* and *Escherichia coli*. As the infection subsided and the wound environment gradually returned to a neutral pH, the nanozyme transitioned to SOD- and CAT-like activities, enabling scavenging of excess ROS and reversal of the pro-inflammatory immune microenvironment. This “on-demand” catalytic switching endowed the nanozyme with microenvironment-responsive therapeutic adaptability, allowing seamless transitions between antibacterial and anti-inflammatory functions throughout different stages of wound healing. Consequently, this strategy represents a promising approach for dynamic and precisely regulated infected wound therapy (Jin et al., 2026). Extending beyond single-metal systems, bimetallic single-atom nanozymes have been developed to further enhance catalytic performance through synergistic effects. For instance, Zhai et al. (2025) developed ultralow-dose Fe–Cu bimetallic single-atom nanozymes which exhibited significantly enhanced POD-like activity and markedly improved photothermal performance compared to single-atom counterparts, owing to the synergistic interaction between Fe and Cu active sites. The enhanced synergistic photothermal–chemodynamic ability of the nanozymes demonstrated strong antibacterial efficacy in both *in vitro* and *in vivo* models, driven by enhanced ROS generation and localized hyperthermia (Zhai et al., 2025). Beyond catalytic therapy alone, single-atom nanozymes have also enabled the integration of additional therapeutic modalities, expanding their functional scope. Mn single-atom nanozymes were incorporated into 3D-printed bioceramic scaffolds, forming an integrated platform with catalytic and therapeutic functionality. Mn nanozymes incorporation ensured POD-like activity while their efficient energy conversion capability enables application in sonodynamic therapy. The combination was used to produce elevated levels of ROS, resulting in effective antibacterial activity against *S. aureus* and *E. coli*. This enhanced antibacterial performance subsequently promoted osteogenic activity, as evidenced by the upregulation of bone-related markers *in vitro*, and improved bone regeneration *in vivo* (Gao et al., 2024).

### 3.5 Structural and mechanical microenvironment engineering

Beyond biochemical antimicrobial strategies, the structural and mechanical characteristics of biomaterials play a fundamental role in shaping the infection microenvironment and regulating tissue regeneration. Parameters such as surface topography and scaffold porosity can be precisely engineered to modulate bacterial adhesion, colonization and survival as well as simultaneously instruct cell behavior and tissue regeneration, ultimately redirecting the race for the surface. In contrast to drug- or ion-based approaches, these strategies rely primarily on physical stimuli that act at the material-microbe and material-host interfaces, influencing bacterial adhesion, immune cell function, the fate of stem cells, vascular growth and matrix deposition. This is particularly relevant in infected tissues, where the very architecture of the biomaterial must both limit microbial persistence and provide a microenvironment conducive to host cell adhesion, migration and tissue organization. Consequently, the structural and mechanical engineering of the microenvironment offers a strategy complementary to biochemical approaches, integrating the design of antimicrobial surfaces with physical guidance to promote regeneration.

#### 3.5.1 Surface topography

Surface topography represents one of the most immediate and influential cues at the biomaterial-microbe interface. It strongly governs microbial adhesion and survival through contact-mediated physical interactions, affecting how bacteria initially attach and subsequently form biofilms (Rizzello et al., 2012; Rizzello et al., 2013). In general, increased roughness often provides more anchoring points for microbes, so implants with sandblasted or micropatterned surfaces tend to accumulate more bacteria than perfectly smooth surfaces. Conversely, engineered antifouling topographies that minimize effective contact area can reduce adhesion: by creating nanoscale valleys or repellent patterns, the number of attachment points drops, and microbial colonization is impaired (Hasan et al., 2013; Hasan and Chatterjee, 2015; Linklater et al., 2021). More recently, periodic arrays of conical nanopillars inspired by natural bactericidal interfaces, such as cicada and dragonfly wings, have been shown not only to deter bacterial adhesion but also to exert bactericidal activity through purely mechanical mechanisms (Ivanova et al., 2012; 2013; Hasan et al., 2013). These nanostructured surfaces can physically rupture bacterial cells upon contact. However, their effectiveness is strongly dependent on bacterial cell wall architecture, showing higher activity against Gram-negative bacteria, while Gram-positive species are generally more resistant to lysis (Ivanova et al., 2012; Hasan and Chatterjee, 2015). These surfaces typically contain nanopillar arrays with heights of 100–300 nm and high aspect ratios that generate mechanical stress on bacterial envelopes upon contact, leading to cell rupture (Ivanova et al., 2012; Hasan et al., 2013; Linklater et al., 2021). Although bacterial envelope deformation and penetration are often considered the primary mechanisms of bactericidal activity, several studies indicate that membrane deformation does not necessarily result in rupture or cell lysis. This suggests that mechanical damage alone may not be able to completely explain the reduced bacterial viability. Instead, oxidative stress and ROS levels were found significantly elevated in *S. aureus* and *E. coli* on nanopillar surfaces, suggesting topography-mediated activation of intracellular pathway terminating in ROS production, which may contribute to impaired bacterial survival (Jenkins et al., 2020). While extensive efforts have focused on leveraging surface topography to limit bacterial adhesion and biofilm formation, these same structural cues are equally critical in regulating host cell behavior and promoting tissue regeneration (Sun et al., 2016; Di et al., 2023). Cells sense micro- and nanoscale features via integrin-mediated adhesions and cytoskeletal tension (Sun et al., 2016; Di et al., 2023), which in turn guide migration, proliferation and differentiation. As a result, a patterned scaffold can encourage stem cells to spread and commit to osteogenesis (for bone repair) or to form tight endothelial junctions (for vascularization), depending on the feature size and arrangement (Sun et al., 2016; Di et al., 2023). Despite the parallel advancement of antimicrobial surface topographies and topography-driven strategies to modulate host cell behavior, relatively few studies have simultaneously addressed both bacterial inhibition and tissue regeneration, particularly through purely physical and structural approaches. This reflects a fundamental design challenge: features that inhibit bacterial adhesion may not always support optimal cell attachment, and *vice versa*. In this context, hierarchical topography, defined as a surface architecture that integrates features across multiple length scales, typically combining micro- and nanoscale structures, has emerged as a promising strategy to generate complex, multi-level physical cues capable of modulating biological interactions. For instance, Alameda et al., engineered hierarchical micro–nano topographies to both direct hMSC behavior and inhibit bacterial adhesion (Figure 5A) (Alameda et al., 2025). Such hierarchical topographies have been fabricated using commonly employed implant polymers, including hard and soft polydimethylsiloxane and poly (methyl methacrylate). Bioinspired moth-eye nanocones in the 10–100 nm range were used for their antibacterial activity against *E*. *coli* and *S*. *aureus* (Figure 5B), whereas micrometer-scale features were selected to regulate MSC response and osteogenic differentiation (Figure 5C) (Alameda et al., 2025). Hierarchical micro- and nanoscale roughness on zirconia surfaces was also obtained using a novel approach that combines ultrashort pulsed direct laser interference patterning with chemical etching. Here, the micropatterns primarily influenced cell shape, alignment, and migration, while the added nanotopography promoted focal adhesion formation and altered cell-surface interactions. Antibacterial performance varied depending on both surface structure and bacterial species: micropatterns limited *P. aeruginosa* colonization through spatial confinement, whereas the nanotopography introduced by etching reduced *S. aureus* adhesion by minimizing effective contact points. In co-culture conditions, hierarchical patterned substrates promoted hMSC adhesion, migration, osteogenic differentiation, and mineralization, while also reducing bacterial colonization and supporting cell viability under infection-like conditions (Garcia-de-Albeniz et al., 2026; Garcia-de-Albeniz et al., 2026). Similarly, hierarchical micro/submicro/nano-scale topographies have been engineered on Ti using different approaches (e.g., sandblasting followed by primary and secondary acid etching), showing enhanced osteogenic commitment of pre-osteoblasts and antibacterial activity against *E. coli* and *S. aureus* (Huang et al., 2014). Chemical functionalization was also employed to further favor cell adhesion on Ti substrates. Indeed, a poly (ethyl acrylate) (PEA) coating on Ti nanowire was used to induce fibronectin unfolding, exposing integrin-binding domains that improve cell attachment, while simultaneously presenting BMP2 (bone morphogenetic protein 2) in a solid-phase configuration to promote synergistic signaling between integrins and growth factors (Damiati et al., 2022; Cuahtecontzi Delint et al., 2024). Relevantly, the coated nanowires were shown to protect MSCs from harmful bacterial quorum sensing molecules in a novel co-culture system of MSCs and *P*. *aeruginosa* (Damiati et al., 2022), inducing MSCs secretion of metabolites, notably citrate, that reduce biofilm formation while helping MSCs differentiation (Figure 5D) (Cuahtecontzi Delint et al., 2024). Another study, nanopatterned substrates were combined with fluoride ion doping. Here, by controlling the hydrolysis of α-tricalcium phosphate using biomimetic and hydrothermal methods, authors produced calcium-deficient hydroxyapatite nanoneedle structures, with their morphology and biological performance tuned via fluoride incorporation. Fluoride incorporation significantly modified nanoneedle size and spacing and enhanced bactericidal activity, particularly against *P*. *aeruginosa*, and to a lesser extent *S*. *aureus*. While fluoride doping alone did not exhibit antibacterial effects, its combination with nanotopography resulted in a synergistic increase in antimicrobial efficacy. Importantly, these surfaces supported the proliferation and osteogenic differentiation of SaOS-2 cells. Co-culture experiments simulating pre- and post-implantation infection scenarios showed strong cell attachment alongside a marked reduction in bacterial colonization (Arca-Garcia et al., 2026). Nanostructured substrates have also been used for gradual release of biocompatible antimicrobial compounds. In a recent study, Ishak et al., functionalized a stem-cell-compatible antimicrobial peptide onto a nanospike surface by physical adsorption. Functionalization with peptide inhibited *S*. *aureus* and *Staphylococcus epidermidis* growth on the nanospike surface, with the topography-induced membrane deformation ultimately increasing surface area for peptide membrane entrance (Ishak et al., 2023). Micrometer-scale surface topography has also been used to probe the complex interplay between biomaterials, macrophages, and bacteria (Xu et al., 2025). To this extent, PDMS square-shaped wells were fabricated using soft lithography to systematically vary both well size and spacing. Square-shaped features with a 5 µm side length and 10 µm depth exhibited up to a 100-fold decrease in phagocytosis compared to flat controls (Xu et al., 2025). Such findings suggest that poorly designed topographies may inadvertently impair immune function and facilitate biofilm formation, rendering them more resistant to host immune responses and antibiotic treatments.

**FIGURE 5:**
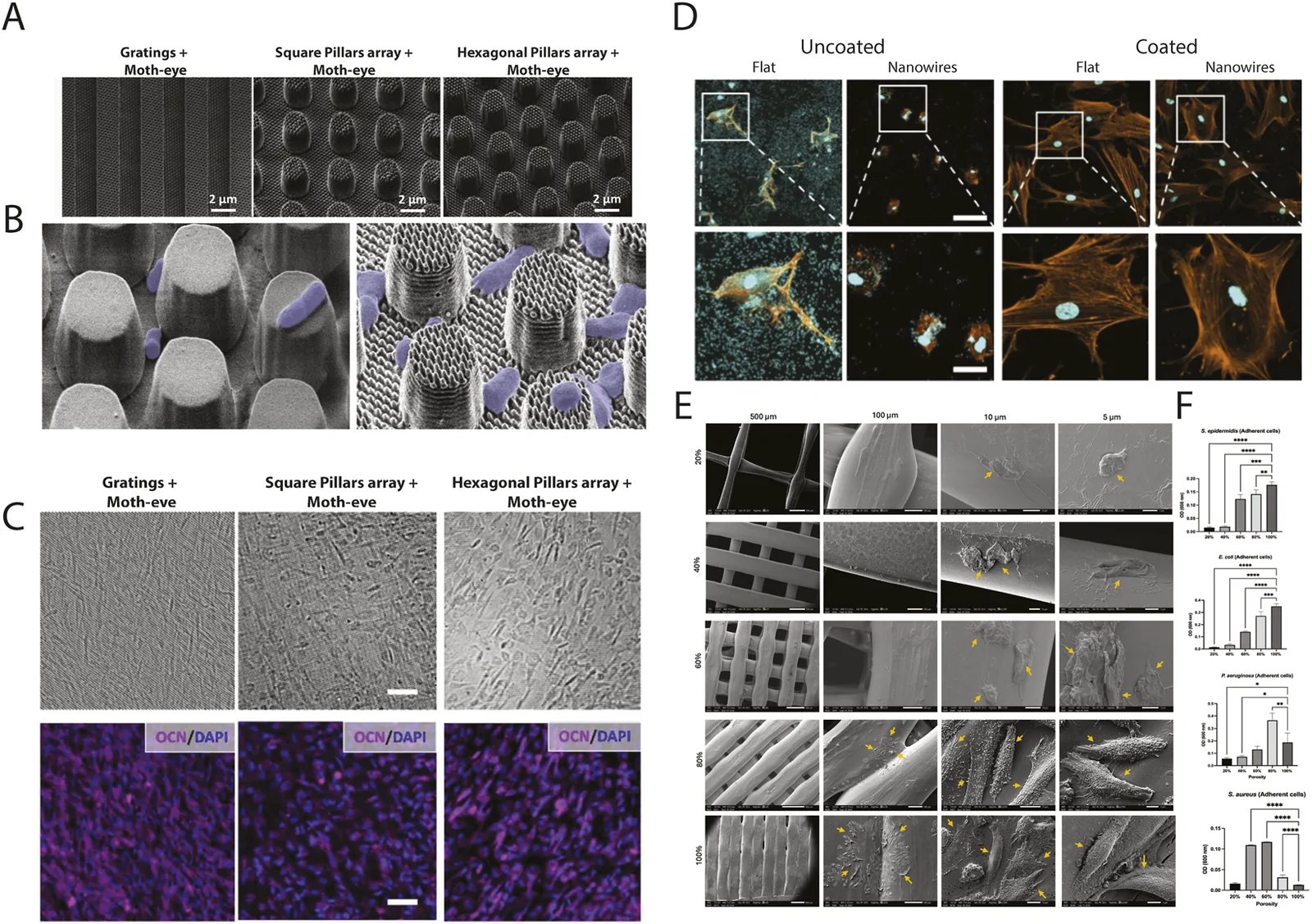
Topography-Driven Modulation of Cellular and Bacterial Responses. **(A)** SEM images of the hierarchical topographies, including hexagonal pillar arrangement, square pillar arrangement, and gratings and moth-eye nanoscale features. **(B)** SEM images of *E. coli* cultured on microtopographies, as well as on the corresponding hierarchical topographies. Scale bar: 1 µm. Adapted from (Alameda et al., 2025) with permission from MDPI. **(C)** Phase-contrast and immunofluorescence images showing nuclei stained with DAPI (blue) and osteocalcin (OCN, magenta), a marker of osteogenic differentiation. Scale bar: 50 µm. **(D)** Fluorescence images showing MSCs nuclei and bacteria stained with DAPI (blue), and actin staining (orange) used to visualize MSCs morphology. Scale bars: 100 μm in the top row and 30 μm in the bottom row. **(E)** SEM images of fibrobasts cultured on PLA scaffolds with different porosities (20%, 40%, 60%, 80%, and 100%). **(F)** Quantification of porosity-dependent bacterial adhesion of *S. epidermidis*, *E. coli*, *P. aeruginosa* and *S. aureus.* Data are presented as mean ± SD. Statistical analysis was performed using one-way ANOVA (*p < 0.05, **p < 0.01, ***p < 0.001, ****p < 0.0001; n = 3). Adapted from (Alqurashi et al., 2025) with permission from Springer Nature.

#### 3.5.2 Porosity

Porosity is a key structural parameter in biomaterials, influencing both bacterial colonization and tissue regeneration by governing mass transport, surface accessibility, and local microenvironmental conditions (Hernandez and Woodrow, 2022; Mukasheva et al., 2024). As such, pore architecture represents a powerful design tool to regulate microbial behavior and host responses. However, achieving this dual functionality requires careful optimization of pore size, interconnectivity, and material composition to balance infection control, tissue integration, and mechanical performance (Hernandez and Woodrow, 2022). From a microbiological perspective, porosity can either promote or hinder bacterial colonization depending on the architecture of the pore network. Bacteria tend to accumulate in protected niches such as pores and cavities, where reduced shear stress and limited fluid exchange favor stable adhesion and biofilm maturation (Hernandez and Woodrow, 2022). For example, on titanium surfaces, Braem et al. (2014) reported increased bacterial adhesion in materials with pore sizes up to 150 μm or porosity above 15%. In contrast, pore size at the nanoscale can have the opposite effect: Feng et al. (2015) demonstrated that alumina surfaces with 15–25 nm pores reduced bacterial attachment, likely due to repulsive forces generated by densely packed pore walls. Moreover, smaller pores and confined environments may also shield bacteria from immune surveillance and antibiotic penetration, contributing to the formation of stagnant environments that support biofilm growth and persistent infections (Hernandez and Woodrow, 2022). While porosity can facilitate bacterial colonization under certain conditions, it also plays a central role in directing host tissue responses. Indeed, pore architecture can be tuned to provide cells with micro- and nanoscale structural cues that regulate adhesion, migration, proliferation, and differentiation. Indeed, interconnected macroporosity (typically > 100 μm) is essential for cell infiltration, vascularization, and nutrient transport (Hernandez and Woodrow, 2022; Mariano et al., 2022; Mukasheva et al., 2024). Furthermore, by controlling the spacing between neighboring anchoring points available to adherent cells, porosity can also be leveraged to control cell–substrate interactions (Mariano et al., 2022). For example, when pore sizes are larger than the cell itself, cells tend to attach to the scaffold surface in a manner similar to that observed on flat, planar substrates. In contrast, pores with dimensions comparable to the cell enable three-dimensional attachment, leading to distinct activation of the mechanotransduction machinery (Mariano et al., 2022). Building on this concept, recent studies have systematically explored the combined effects of porosity on both cell behavior and bacterial colonization. In a recent study, highly porous scaffolds with controlled porosity have been achieved via 3D printing to investigate the combined effects of varying porosity levels (20%–100%) on human skin fibroblast growth and bacterial colonization by Gram-positive (*S. epidermidis*, *S. aureus*) and Gram-negative (*P. aeruginosa*, *E. coli*) species (Alqurashi et al., 2025). Cell studies revealed a time-dependent increase in fibroblasts colonization (Figure 5E), with optimal viability observed at intermediate-to-high porosities (60%–80%). Bacterial adhesion, however, varied depending on species. *S. epidermidis* and *E. coli* showed increased colonization with higher porosity, peaking at 100%, whereas *P. aeruginosa* exhibited maximum adhesion at 80%. In contrast, S. aureus preferentially adhered to scaffolds with intermediate porosity (40%–60%) and less to fully porous structures (Figure 5F) (Alqurashi et al., 2025). *In vivo* biological environments, the interplay between porosity, bacterial colonization and tissue regeneration becomes more complex. While high porosity increases available surface area for bacterial attachment, it also promotes tissue ingrowth, which is one of the most effective defenses against biofilm formation, thereby shifting the balance in the race for the surface toward host tissue integration (Hernandez and Woodrow, 2022). This dual effect has been demonstrated in several studies. Porous implants (100–200 μm pores) were more susceptible to infection if contaminated at or before implantation; however, once tissue integration occurred, these materials became more resistant than dense implants (Hernandez and Woodrow, 2022). Similarly, high-density polyethylene (100–250 μm pores) implants were prone to infection when exposed to *S. aureus* immediately after surgery, but larger-pore materials exhibited greater resistance at later time points due to faster tissue integration (Hernandez and Woodrow, 2022).

Beyond purely structural effects, porosity can also be engineered to enhance functionality through material design strategies. For instance, scaffolds with increased porosity and nanoscale surface features that promote cell attachment have been achieved using a Pickering emulsion templating strategy. Here, the addition of aminated gelatin nanoparticles and copper–hydroxyapatite nanoparticles was employed not only to replicate the native bone composition and ECM environment, but also to create a nanotopographical interface favorable for cell-material interactions, providing abundant anchoring sites for cells (Chu et al., 2025). As a result, the scaffold displayed finely tuned interfacial characteristics, where the nanoparticles contribute an optimal friction coefficient and surface roughness, both essential for improving implant performance. This results in biomimetic, highly porous scaffolds that closely resemble the architectural organization of natural bone, providing a favorable environment that enhances osteoblast function, speeds up bone matrix mineralization, and supports neovascularization. The high porosity further facilitated the controlled release of copper ions from the exposed nanoparticles, contributing to antibacterial activity against *S. aureus, Escherichia coli* and other carbapenem-resistant Enterobacteriaceae as well as angiogenic, and osteogenic functions (Chu et al., 2025). Porosity may also be tuned not only to improve mass transport within biomaterials, facilitating the diffusion of oxygen, nutrients, antimicrobial agents, and immune mediators, but also to regulate the release of compounds with antimicrobial activity. For instance, porous scaffolds fabricated through a mineralization process in which nanocrystalline magnesium-doped apatite was heterogeneously nucleated onto self-assembling collagen fibrils were used for the controlled release of clinically relevant antibiotics (i.e., vancomycin, gentamicin, and tobramycin). These scaffolds, by modulating antibiotic release, showed antibacterial efficacy against *S. aureus* as early as 24 h after treatment. Furthermore, they provided a biomimetic three-dimensional environment, laying a strong foundation for bone regeneration (Tavoni et al., 2025). In another study, Lanno et al., developed chloramphenicol-loaded porous PCL scaffolds. Here, overall scaffold porosity but also nanoscale pores on individual fibers were shown to significantly influence drug release and cell–material interactions, improving fibroblast attachment, migration, and proliferation compared to nonporous micro- and nanofiber scaffolds alone while simultaneously showing strong antibiofilm activity against *E. coli* (Lanno et al., 2020). Despite these advantages, increased porosity is often associated with reduced mechanical strength, highlighting an inherent trade-off in scaffold design. Alqurashi et al. (2025) demonstrated that increased porosity (60%–80%) decreased tensile strength in PLA scaffolds, indicating greater flexibility but reduced mechanical integrity. While this may be advantageous for cell infiltration, it may limit the applications of these scaffolds, preventing their use in load-bearing environments. In this context, gradient-porous scaffolds, in which pore size and/or porosity vary spatially within the device, represent a promising strategy for addressing these conflicting requirements, providing highly porous regions that promote cell infiltration and vascularization, together with denser regions that ensure mechanical stability. Material composition and post-processing strategies can partially compensate for the mechanical limitations associated with high porosity. For example, incorporating inorganic fillers such as hydroxyapatite or bioactive glass can enhance stiffness and compressive strength, while providing bioactive signals for tissue regeneration (Xu D. et al., 2022). Additionally, thermal treatments that increase polymer crystallinity have been shown to recover part of the mechanical performance lost due to porosity (Esposti et al., 2022). In conclusion, porosity emerges as a critical and multifaceted design parameter in biomaterials, simultaneously influencing bacterial colonization and tissue regeneration. Its effects are inherently dualistic: while certain pore architectures may create niches favorable for bacterial adhesion and biofilm formation, appropriately engineered porosity can enhance tissue integration, mass transport, and the delivery of antimicrobial agents. Therefore, the rational design of porous scaffolds requires a delicate balance, optimizing pore size, interconnectivity, and material composition to promote regenerative outcomes while minimizing infection risk. Future efforts should focus on hierarchical, multifunctional architectures that integrate structural, biological, and antimicrobial features to better control the local microenvironment and improve biomaterial performance.

#### 3.5.3 Surface physicochemical properties and interfacial mechanics

In addition to surface topography and porosity, physicochemical and mechanical properties at the biomaterial interface strongly influence both microbial adhesion and host-tissue responses. Surface charge, wettability, and stiffness regulate the initial interaction of bacteria with material surfaces, but they also determine protein adsorption, integrin engagement, cytoskeletal organization, and subsequent tissue integration (Metwally and Stachewicz, 2019; Hu and Zhang, 2021; Li et al., 2021). Thus, these parameters represent important design variables for simultaneously limiting bacterial colonization and establishing a regenerative host interface (Li et al., 2021; Yi et al., 2025). Surface charge is one of the earliest determinants of biological interactions with an implanted material and can be exploited to reduce bacterial adhesion or promote contact-mediated killing (Li et al., 2021; Yi et al., 2025). Negatively charged surfaces may limit nonspecific bacterial and protein adsorption through electrostatic repulsion and the formation of strongly hydrated interfacial layers, whereas positively charged surfaces can enhance bacterial capture and membrane disruption when appropriately engineered (Li et al., 2021; Yi et al., 2025). Zwitterionic surfaces provide an alternative strategy by combining cationic and anionic groups within the same interface. These surfaces can strongly bind water molecules through ionic solvation, generating a stable hydration layer that acts as both an energetic and physical barrier to protein adsorption and subsequent bacterial attachment (Li et al., 2021; Yi et al., 2025).

Similarly, surface charge also affects adsorption and conformation of extracellular matrix proteins, which subsequently regulate host-cell attachment (Stephansson et al., 2002; Keselowsky et al., 2003; Rapuano et al., 2012). In addition to surface topography and porosity, physicochemical and mechanical properties at the biomaterial interface strongly influence both microbial adhesion and host-tissue responses. Surface charge, wettability, and stiffness regulate the initial interaction of bacteria with material surfaces, but they also determine protein adsorption, integrin engagement, cytoskeletal organization, and subsequent tissue integration (Metwally and Stachewicz, 2019; Hu and Zhang, 2021; Li et al., 2021). Thus, these parameters represent important design variables for simultaneously limiting bacterial colonization and establishing a regenerative host interface (Li et al., 2021; Yi et al., 2025). Surface charge is one of the earliest determinants of biological interactions with an implanted material and can be exploited to reduce bacterial adhesion or promote contact-mediated killing (Li et al., 2021; Yi et al., 2025). Negatively charged surfaces may limit nonspecific bacterial and protein adsorption through electrostatic repulsion and the formation of strongly hydrated interfacial layers, whereas positively charged surfaces can enhance bacterial capture and membrane disruption when appropriately engineered (Li et al., 2021; Yi et al., 2025). Zwitterionic surfaces provide an alternative strategy by combining cationic and anionic groups within the same interface. These surfaces can strongly bind water molecules through ionic solvation, generating a stable hydration layer that acts as both an energetic and physical barrier to protein adsorption and subsequent bacterial attachment (Li et al., 2021; Yi et al., 2025).

Similarly, surface charge also affects adsorption and conformation of extracellular matrix proteins, which subsequently regulate host-cell attachment (Stephansson et al., 2002; Keselowsky et al., 2003; Rapuano et al., 2012). For example, a titanium implant self-powered with a triboelectric nanogenerator was used to harvest and convert mechanical energy generated by daily human motions into electrical energy to establish a negatively charged surface that effectively inhibits bacterial adhesion, reduces bacterial load and biofilm formation, while simultaneously enhancing osteogenesis (Shi et al., 2020). Similarly, zwitterionization of 3D Ti6Al4V scaffolds markedly reduced bacterial adhesion and biofilm formation while preserving biocompatibility, bioactivity, and host-cell adhesion, supporting their potential for bone-regenerative applications (Rodriguez-Palomo et al., 2016).

Importantly, the appropriate surface charge is highly dependent on the intended clinical application: positively charged contact-killing coatings may be effective for orthopedic implants but are generally less appropriate for blood-contacting devices such as vascular catheters, where adsorption of fibrin and platelets onto charged surfaces may promote thrombus formation and potentially lead to occlusion. Conversely, anti-biofouling coatings that minimize the adhesion of bacteria, proteins, and cells are particularly attractive for catheter applications. However, the same low-adhesion properties can be detrimental for orthopedic implants, where stable cell attachment and tissue integration are essential for long-term fixation (Stephansson et al., 2002; Metwally and Stachewicz, 2019; Li et al., 2021; Crago et al., 2024).

More recently, charge-convertible implant coatings have been used to dynamically tune antibacterial performance and cell adhesion for different biomedical applications, including orthopedic implants and vascular catheters. Notably, a Cu^2+^-loaded, positively charged coating based on a copolymer containing pendant carboxybetaine tert-butyl ester groups was fabricated on the surface of titanium implants. The positively charged coating exhibited potent and sustained *in vitro* antibacterial activity through the synergistic effects of Cu^2+^-mediated bacterial killing and contact-active antimicrobial mechanisms. In addition, the coating demonstrated excellent cytocompatibility, supported favorable cell adhesion and proliferation, promoted osteogenic differentiation, and reduced inflammatory responses while enhancing tissue healing in an *in vivo* orthopedic implantation model, highlighting its suitability for tissue-integrating implants (Li S. et al., 2023).

Following acid treatment, the positively charged coating was readily converted into an electrically neutral zwitterionic coating. The resulting surface retained strong and long-lasting *in vitro* antibacterial activity through the combined effects of Cu^2+^-mediated killing and antifouling mechanisms, while reducing nonspecific cell adhesion, making it particularly advantageous for implantable devices that do not require tissue integration, such as vascular catheters, where minimizing biological fouling is desirable (Li S. et al., 2023).

Surface wettability similarly influences both bacterial colonization, cell attachment and regenerative behavior (Li et al., 2021; Rajaramon et al., 2023; Crago et al., 2024; Lin et al., 2024; Yi et al., 2025). Superhydrophilic surfaces, can resist bacterial attachment by maintaining a strongly hydrated interface that limits direct contact between microorganisms and the underlying material. Similarly, superhydrophobic surfaces inspired by naturally self-cleaning interfaces such as lotus leaves, can deter bacterial colonization. Materials including SiO_2_, CuO, TiO_2_, polydimethylsiloxane, polypyrrole, and polyvinylidene fluoride have been used to generate superhydrophobic interfaces capable of trapping an air layer and reducing the effective contact area between the material, surrounding liquid, and bacterial cells (Li et al., 2021; Yi et al., 2025). From a regenerative perspective, however, both hydrophilicity and persistent superhydrophobicity may reduce protein adsorption and host-cell attachment. The optimal wetting state therefore depends on the intended application and may require a compromise between minimizing initial bacterial adhesion and supporting subsequent cellular colonization, vascularization, and extracellular matrix formation. This trade-off has pushed the development of multifunctional interfaces in which hydrophilic antibacterial components, such as PEG, are combined with cell-selective adhesive ligands. For example, RGD-functionalized PEG-based coatings have been shown to preserve substantial resistance to bacterial adhesion while restoring osteoblast attachment and spreading, illustrating how selective ligand presentation can reconcile antibacterial activity with regenerative cell–material interactions (Chua et al., 2008; Maddikeri et al., 2008; Hoyos-Nogués et al., 2018). Dinamically tunable or switchable wetting interfaces have also been developed. Děkanovský et al. (2019) for instance, developed an electrically responsive polymeric surface that initially maintained a superhydrophobic state and an interfacial air layer but could subsequently switch toward a hydrophilic state under electrical stimulation, enabling release of an antibacterial agent after bacterial attachment. More broadly, such temporal control offers an attractive strategy for regenerative biomaterials: an initially anti-adhesive or bactericidal surface could reduce microbial colonization during the high-risk early period after implantation, followed by transition toward a more cell-permissive interface that supports host-cell adhesion, migration, matrix deposition, and long-term integration (Zhou et al., 2023; Zhou M. et al., 2024; Zhang et al., 2024). For instance, a smart titanium surface combining a PEG-based thermoresponsive polymer with the antimicrobial peptide HHC36 was developed to dynamically regulate antibacterial and regenerative functions. The coating reduced bacterial adhesion and biofilm formation, while temperature-induced polymer collapses increased surface hydrophobicity, exposed antimicrobial peptides, and enhanced protein and host-cell adhesion. This adaptive behavior supported stem-cell protection and osteogenic activity while strengthening antibacterial performance. In rabbit subcutaneous and bone-defect infection models, the surface reduced infection and promoted tissue healing, demonstrating how dynamic surface engineering can balance antimicrobial activity with host-cell integration (Zhou et al., 2023).

Interfacial stiffness provides another layer of biological regulation. Because bacterial species differ in cell-wall structure, shape, and motility, their response to substrate mechanics is not uniform. For example, flagellated Gram-negative *E. coli* and non-motile Gram-positive *S. aureus* can exhibit distinct adhesion behaviors on substrates of different stiffness (Kolewe et al., 2015; Straub et al., 2019).

Several studies have reported greater bacterial attachment to stiffer materials, indicating that mechanical properties can modulate attachment independently of major changes in surface chemistry (Lichter et al., 2008; Xiao et al., 2024; Riedel et al., 2025). However, this relationship is not universal. For instance, *S. aureus* adhesion has been reported to decrease on softer hydrogels (Kolewe et al., 2015). These apparently conflicting findings indicate that the effect of stiffness on bacterial adhesion depends on multiple factors, including bacterial species, morphology and motility, material composition and viscoelasticity (Lichter et al., 2008; Xiao et al., 2024; Riedel et al., 2025). Beyond its effects on microorganisms, interfacial stiffness is also a major regulator of host-cell behavior. Substrate stiffness regulates focal adhesion maturation, cytoskeletal tension, migration, proliferation, and differentiation, and can therefore influence whether the surrounding tissue develops toward fibrotic encapsulation or constructive regeneration (Di et al., 2023; Saraswathibhatla et al., 2023). For mesenchymal stromal cells, mechanical properties can influence lineage commitment, whereas endothelial cells and fibroblasts respond to stiffness through changes in migration, matrix production, and organization (Saraswathibhatla et al., 2023). Although relatively few studies have isolated stiffness as the sole determinant of both antibacterial and regenerative performance, these findings suggest that mechanical tuning may provide a complementary, drug-free strategy for balancing resistance to bacterial colonization with tissue-specific regenerative responses.

Overall, surface charge, wettability, and stiffness represent interconnected interfacial cues that regulate both bacterial behavior and host-cell responses. Because optimization of a given property for antimicrobial activity may not necessarily favor tissue integration, these parameters should be considered collectively and tailored to the intended tissue and clinical application. Within the broader “race for the surface” framework, successful biomaterial design should therefore aim not simply to minimize bacterial attachment, but to selectively suppress microbial colonization while establishing an interface that supports host-cell adhesion, immune regulation, vascularization, matrix remodeling, and long-term tissue integration.

## 4 Outlook and future directions

The development of multifunctional biomaterials marks a significant shift in the design of anti-infective strategies for tissue regeneration. Rather than acting solely as passive barriers to bacterial colonization or reservoirs for antimicrobial agents, next-generation biomaterials are increasingly conceived as active therapeutic interfaces capable of simultaneously controlling infection, regulating inflammation, and promoting tissue regeneration. As summarized in Figure 6, the major biomaterial platforms discussed in this review act through distinct but complementary mechanisms, integrating antimicrobial activity with immunomodulation, angiogenesis, extracellular matrix remodeling, and tissue-regenerative functions, while presenting specific advantages and limitations. This evolution reflects a broader recognition that bacterial eradication alone is not sufficient to restore tissue function. In infected wounds, implant-associated infections, and chronic inflammatory microenvironments, microbial persistence is tightly linked to immune dysregulation, oxidative stress, hypoxia, pH imbalance, ECM degradation, and impaired cellular regeneration.

**FIGURE 6:**
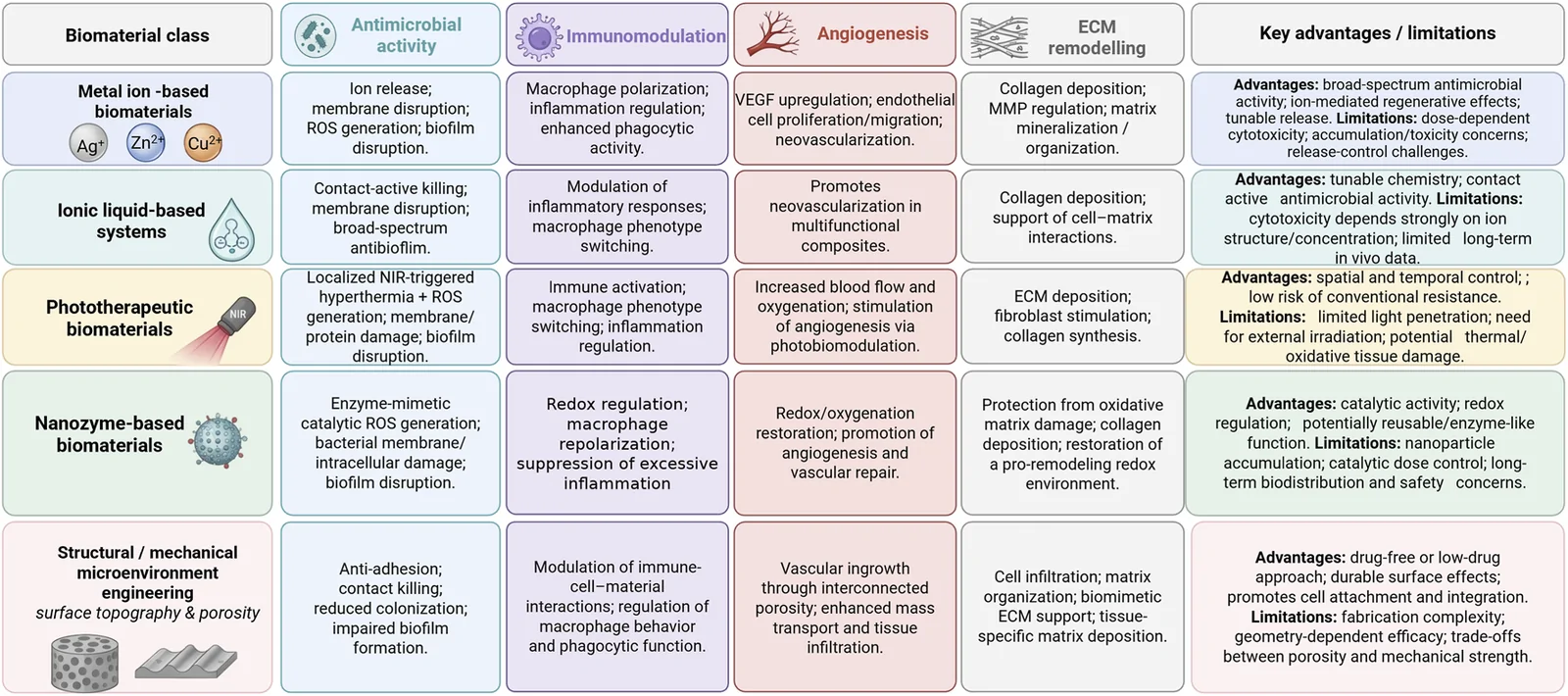
Mechanistic summary of multifunctional biomaterial platforms for infection control and tissue regeneration. The figure compares the major biomaterial classes discussed in this review, including metal ion-based biomaterials, ionic liquid-based systems, phototherapeutic biomaterials, nanozyme-based biomaterials, and structural/mechanical microenvironment engineering. For each platform, the principal mechanisms contributing to antimicrobial activity and tissue regeneration are summarized, together with the main advantages and limitations associated with each approach. Created in BioRender. Mariano, G. (2026) BioRender.com/8q9njdo.

Accordingly, the central design principle emerging from this field is that effective biomaterial platforms must address the microbial and host components of the pathological niche in a coordinated manner.

Despite rapid progress, several translational challenges continue to limit clinical advancement (Table 2). Many multifunctional biomaterials are still evaluated in simplified *in vitro* systems or short-term small-animal models. Future studies should include standardized antibacterial and antibiofilm assays, clinically relevant polymicrobial biofilm models, dynamic co-culture systems that include immune and stromal cells, long-term biocompatibility testing, degradation and clearance analyses, and rigorous evaluation of immunological and regenerative outcomes. A further limitation is the mismatch between commonly used acute rodent wound-infection models and the clinical complexity of implant-associated infections. Although wound models are valuable for initial proof-of-concept studies, they do not fully reproduce the peri-implant environment, where foreign material, damaged or poorly perfused tissue, persistent mature biofilms, bone involvement, and complex inflammatory and immunosuppressive responses can promote bacterial persistence. More clinically representative models are therefore needed to determine whether antimicrobial and regenerative efficacy is maintained (Carli et al., 2017; López-Torres et al., 2020; Chen et al., 2025). For example, murine periprosthetic joint infection models incorporating load-bearing titanium implants can reproduce peri-implant bacterial persistence, mature biofilm formation, inflammatory responses, impaired osseointegration, and septic implant loosening, thus providing a more clinically relevant platform for evaluating biomaterial performance (Carli et al., 2017).

**TABLE 2:** Translational challenges, potential solutions, and future perspectives for multifunctional biomaterials**.** The table summarizes the main barriers to clinical translation, together with potential strategies to address these challenges and priorities for future development.

Current challenge	Potential solutions	Future perspectives
Limited clinical relevance of experimental models	Adopt polymicrobial biofilm models, dynamic co-cultures containing immune and stromal cells, and disease-relevant large-animal models	Develop standardized preclinical pipelines that better reproduce the temporal and biological complexity of infected wounds and implant-associated infections
Long-term safety and biocompatibility	Conduct extended cytotoxicity, immunotoxicity, genotoxicity, hemocompatibility, and local and systemic toxicity studies	Define clinically relevant safety thresholds and long-term benefit–risk profiles for each material class and application
degradation, clearance, and biodistribution	Characterize degradation products, tissue accumulation, organ distribution, metabolic fate, and excretion using quantitative *in vivo* methods	Design fully degradable materials with predictable clearance and minimal long-term retention
Potential toxicity of biologically active components	Minimize concentrations of metal ions, ionic liquids, photothermal agents, and nanozymes while maintaining therapeutic efficacy. Incorporate spatially and temporally controlled activation	Establish material-specific dose–response relationships and safe therapeutic windows under clinically relevant conditions
Manufacturing scalability and batch reproducibility	Develop simplified, robust, and scalable synthesis methods; define critical process parameters and critical quality attributes; and introduce in-process quality controls	Implement good manufacturing practice-compatible production while preserving composition, architecture, release kinetics, and biological performance during scale-up
Sterilization and storage stability	Evaluate the effects of irradiation, heat, filtration, and chemical sterilization on material structure and function. Perform accelerated and long-term stability testing	Develop sterilization-compatible formulations with clinically practical shelf lives, packaging, and storage conditions
Regulatory complexity and classification	Define the primary mode of action early and determine whether the platform should be regulated as a device, drug, biologic, or combination product. Engage regulatory authorities during development	Establish clearer regulatory pathways and evaluation frameworks for multifunctional and responsive biomaterial systems
Commercialization and cost-effectiveness	Simplify material composition, reduce manufacturing steps, select accessible raw materials, and compare performance and cost with current standards of care	Develop commercially viable platforms that provide measurable clinical benefits without excessive production or implementation costs
Integration into clinical workflows	Consider administration, implantation, activation requirements, treatment duration, clinician training, patient acceptability, and compatibility with existing procedures	Prioritize application-specific designs that can be readily incorporated into wound-care, surgical, and implant-management pathways
Environmental impact	Reduce the use of persistent metals and synthetic ionic components; adopt low-dose, degradable, recyclable, or renewable material strategies. Assess environmental release throughout the product life cycle	Integrate sustainability and life-cycle assessment into biomaterial development and regulatory evaluation
Translation of engineered living materials	Establish control over cell or microorganism viability, containment, genetic stability, immune compatibility, and biosafety. Incorporate fail-safe or self-limiting mechanisms	Develop safe, feedback-regulated living systems that sense pathological cues and adapt therapeutic output to changing infection and healing conditions

Moreover, persistent *S*. *aureus* infection in such models is characterized not only by sustained inflammatory responses but also by activation of inhibitory immune pathways and impaired T-cell responses, highlighting immunological features that are not adequately captured by simple wound-infection models (Sokhi et al., 2022). Future preclinical validation should therefore adopt a staged approach in which promising materials progress from simplified screening models to disease-specific implant infection models that more closely reproduce the intended clinical setting (Li et al., 2021; Chen et al., 2025).

It will also be important to distinguish between accelerated wound closure and true tissue regeneration, which should include the assessment of vascularization, matrix organization, innervation, epithelial or bone maturation, mechanical integrity, and functional recovery. For implant applications, osseointegration, fibrous encapsulation, inflammatory response, and resistance to recurrent infection should be assessed over clinically meaningful timeframes. Future studies should also consider the temporal dynamics of immunomodulation, since excessive suppression of early inflammation may compromise microbial clearance, whereas prolonged inflammatory activation can impair tissue regeneration.

Material safety and manufacturability will also determine clinical translation. Systems based on metal ions, ionic liquids, photothermal agents, and nanozymes require careful optimization of dose, degradation products, biodistribution, and long-term toxicity. The same properties that make these materials biologically active, such as redox activity, membrane interactions, ionic conductivity, or catalytic ROS generation, may also raise safety concerns if not precisely controlled. Scalable synthesis, sterilization stability, batch reproducibility, regulatory classification, and storage stability must therefore be considered early in the design process.

Regulatory approval may be particularly challenging for multifunctional platforms that combine material, drug, device, or biological components, as their classification, quality-control requirements, and evaluation pathways may differ across jurisdictions. Successful commercialization will therefore require early engagement with regulatory authorities, clearly defined critical quality attributes, standardized manufacturing protocols, and robust evidence of safety, efficacy, and mechanism of action. Manufacturing strategies must also be compatible with scale-up while preserving material composition, architecture, release behavior, and biological performance across production batches. In parallel, clinical translation will depend on the use of clinically relevant disease models, comparison with current standards of care, practical routes of administration or implantation, cost-effectiveness, and the feasibility of integration into existing healthcare workflows. In addition, the environmental impact of antimicrobial materials, particularly those containing persistent metals or synthetic ionic components, should be addressed through degradable, low-dose, and sustainable material strategies. Future development should therefore consider regulatory feasibility, commercial viability, manufacturing scalability, reproducibility, and environmental sustainability alongside biological efficacy from the earliest stages of material design.

Finally, future advances will likely arise from interdisciplinary integration. The most effective platforms will not simply combine multiple active agents, but will rationally coordinate antimicrobial, immunological, mechanical, electrical, biochemical, and regenerative cues within a single material architecture. Along this trajectory, engineered living materials can be viewed as an advanced extension of responsive biomaterials, in which sensing and therapeutic functions are not only encoded in the material chemistry but are also mediated by living or cell-derived components. By incorporating living cells, microorganisms, or bioactive cell-derived components within material matrices, these platforms could be designed to detect infection-associated cues, respond to changes in pH, oxygen tension, inflammatory mediators, or bacterial metabolites, and locally produce antimicrobial, immunomodulatory, angiogenic, or regenerative signals. This dynamic, feedback-regulated behavior could be particularly valuable in pathological microenvironments that evolve over time, such as chronic wounds, implant-associated infections, and complex tissue defects, where microbial control, immune regulation, vascularization, and matrix remodeling must be temporally coordinated. In this sense, engineered living materials offer the possibility of moving beyond predefined release profiles toward adaptive therapeutic systems capable of adjusting their activity according to the stage of infection and healing. However, their clinical translation will require careful control of viability, containment, genetic stability, immune compatibility, and biosafety.

Overall, the future of antimicrobial biomaterials lies in the development of systems that are selective, adaptive, and regenerative. The next-generation of materials should not only prevent or eliminate infection, but also actively guide the biological sequence of healing.

Achieving this goal will require the rational integration of infection control, immune regulation, microenvironmental remodeling, and tissue-specific regeneration, together with rigorous validation in clinically relevant models. By addressing these interconnected priorities, multifunctional biomaterials have the potential to redefine therapeutic strategies for wounds, implants, and complex tissue defects.

## Glossary

ALP: Alkaline phosphatase
BG: Bioactive glass
BMP-2/BMP2: Bone morphogenetic protein 2
BP: Black phosphorus
CAT: Catalase
CDT: Chemodynamic therapy
DOPA: 3,4-Dihydroxy-L-phenylalanine
ECM: Extracellular matrix
EPS: Extracellular polymeric substance
GelMA: Gelatin methacryloyl
GOD: Glucose oxidase
HIF-1α: Hypoxia-inducible factor 1-alpha
HUVECs: Human umbilical vein endothelial cells
IL: Ionic liquid
iNOS: Inducible nitric oxide synthase
MBG: Mesoporous bioactive glass
MIC: Mminimum inhibitory concentration
MMPs: Matrix metalloproteinases
MOF: Metal–organic framework
MRSA: Methicillin-resistant *Staphylococcus aureus*
MSCs: Mesenchymal stromal/stem cells
NIR: Near-infrared
NO: Nitric oxide
OCN: Osteocalcin
OPN: Osteopontin
OXD: Oxidase
PCL: Polycaprolactone
PDA: Polydopamine
PDMS: Polydimethylsiloxane
PDT: Photodynamic therapy
PEA: Poly(ethyl acrylate)
PEG: Polyethylene glycol
PIA: Polysaccharide intercellular adhesin
PLA: Polylactic acid
PLLA: Poly-L-lactic acid
POD: Peroxidase
PPy: Polypyrrole
PTT: Photothermal therapy
PVA: Poly(vinyl alcohol)
ROS: Reactive oxygen species
SEM: Scanning electron microscopy
SOD: Superoxide dismutase
TEM: Transmission electron microscopy
VEGF: Vascular endothelial growth factor
vWF: von Willebrand factor
ZIF-8: Zeolitic imidazolate framework-8
